# Small interfering RNAs generated from the terminal panhandle structure of negative-strand RNA virus promote viral infection

**DOI:** 10.1371/journal.ppat.1012789

**Published:** 2025-01-03

**Authors:** Wan Zhao, Qiong Li, Mengqi Sun, Lan Luo, Xiaoming Zhang, Feng Cui

**Affiliations:** 1 State Key Laboratory of Integrated Management of Pest Insects and Rodents, Institute of Zoology, Chinese Academy of Sciences, Beijing, China; 2 CAS Center for Excellence in Biotic Interactions, University of Chinese Academy of Sciences, Beijing, China; Agriculture and Agri-Food Canada, CANADA

## Abstract

Virus-derived small interfering RNAs (vsiRNAs) have been widely recognized to play an antiviral immunity role. However, it is unclear whether vsiRNAs can also play a positive role in viral infection. Here, we characterized three highly abundant vsiRNAs mapped to the genomic termini of rice stripe virus (RSV), a negative-strand RNA virus transmitted by insect vectors. The three vsiRNAs shared 11 nucleotides due to the conservative genomic termini and were likely generated from viral terminal panhandle structure, depending on both Dicer1 and Dicer2 in insects. In addition to targeting viral RNAs in a miRNA-like manner, the three vsiRNAs coordinately downregulated the expression of *DOPA decarboxylase*, thereby suppressing the prophenoloxidase immune reaction in insect vectors. In vsiRNA-silenced transgenic rice, the viral titer significantly decreased, indicating that these vsiRNAs promote RSV replication in rice. This study elucidates a unique function of vsiRNAs derived from the conserved panhandle structure of negative-strand RNA viruses in enhancing viral infection.

## Author summary

RNA interference (RNAi) is one of the important innate immune systems against viruses in insects. Virus-derived small interfering RNAs (vsiRNAs) have been widely recognized to play an antiviral immunity role. Here, we find that three vsiRNAs generated from the terminal panhandle structure of a rice virus promote viral infection in both insect vectors and host plants. These vsiRNAs coordinately target *DOPA decarboxylase* to inhibit melanization immune reaction in insect vectors. The vsiRNA-silenced transgenic rice exhibits an antiviral phenotype. Our work reveals a conserved positive effect of vsiRNAs from negative-strand RNA viruses on viral infection.

## Introduction

RNA interference (RNAi) is a ubiquitous cellular process that regulates mRNA stability and translation in most eukaryotes, including nematodes, arthropods, vertebrates, and plants [1]. The RNAi process is driven by small RNAs of 20 to 31 nucleotides (nt), including small interfering RNAs (siRNAs), microRNAs (miRNAs), and piwi-interacting RNAs (piRNAs) [2]. The generation of miRNAs and siRNAs is dependent on the RNase III-type enzymes such as Dicer or Dicer-like (DCL) [3]. Mature miRNAs and siRNAs are loaded into Argonaute (AGO) proteins, forming RNA-induced silencing complexes (RISCs) for posttranscriptional regulation [3]. siRNAs perfectly base-pair with their targets, leading to their cleavage and degradation [4]. Mechanistic action of miRNAs differs largely between plants and animals (including insects). In plants, miRNAs typically target mRNAs with perfect or near-perfect complementarity, leading to endonucleolytic cleavage of mRNA occurring between the 10^th^ and 11^th^ nucleotides of the miRNA [3,5]. In contrast, the target recognition in animals and insects is primarily mediated by the seed region, comprising bases 2-8, of the miRNA, the rest of which pairs imperfectly with the target [5]. In this case, miRNAs usually silence gene expression via deadenylation-dependent mRNA decay, in which the target mRNA is degraded by the enzymes involved in the classical cellular 5’-to-3’ mRNA decay pathway [6,7].

RNAi plays an important innate immune role against viruses in plants, animals, and insects [8–10]. In addition to host small RNAs, viruses hijack the host RNAi system to produce viral siRNAs (vsiRNAs) or miRNAs (vmiRNAs) [9,11]. vsiRNAs are derived from viral double-stranded RNA replication intermediates or the structural features of the viral single-stranded RNA [12]. They play a classical RNAi antiviral role by cleaving and degrading viral RNAs [12,13]. vmiRNAs are typically produced from the 3’ or 5’ terminal untranslated regions (UTRs) of DNA viruses and positive-strand RNA virus [14]. The functions of vmiRNAs in viral infection are diverse. vmiRNAs can inhibit or facilitate viral replication by targeting host mRNAs or viral RNAs in a miRNA-like manner [15–17]. For instance, Influenza H5N1 virus-specific vmiRNA, namely, miR-HA-3p, increases antiviral cytokine production by suppressing host *PCBP2* expression [15]. KUN-miR-1, encoded by West Nile virus, upregulates *GATA4* mRNA to facilitate virus replication in mosquito cells [16]. Dengue virus (DENV) derived vmiRNA, DENV-vsRNA-5, inhibits DENV replication by targeting viral nonstructural protein 1 gene [17]. Additionally, vsiRNAs also function through a miRNA-like mechanism. Wheat yellow mosaic virus (WYMV)-derived vsiRNA1 has been found to activate host immunity by suppressing the expression of wheat *TaAAED1* in a miRNA-like manner [18]. However, it remains unclear whether vsiRNAs play a positive role in viral infection by regulating host genes.

The 3’ termini of all RNA segments from the segmented, negative-strand RNA viruses are highly conserved in the terminal 10 nt and partially complementary with the 5’ termini to form a panhandle structure, which is essential for initiation of viral RNA synthesis [19–21]. Rice stripe virus (RSV) is a typical negative-strand RNA virus of the *Tenuivirus* genus and is efficiently transmitted by the small brown planthopper *Laodelphax striatellus* in a persistent-propagative mode [22]. As an insect-transmitted plant virus, it is crucial to maintain a balance between virus load and the antiviral immune response to avoid pathogenicity in insect vectors [23]. RSV contains four single-stranded genomic RNA segments encoding one nucleocapsid protein (NP), one RNA-dependent RNA polymerase (RdRp), and five nonstructural proteins [24, 25]. Each RNA segment contains ~20 conserved nucleotides at the 3’- and 5’-ends [26]. Our previous work has shown that the terminal regions of RSV RNA segments are the hot spots of vsiRNA production [27]. Considering the sequence conservation in the genomic termini of negative-strand RNA viruses, the vsiRNAs derived from viral terminal regions function synchronously and have special influences on viral infection.

In this study, we characterized three highly abundant vsiRNAs mapped to the 3’ terminal UTRs of the complementary genomic RNAs of RSV and explored their biogenesis and biological functions during viral infection in insect vectors and host plants.

## Results

### Characterization of vsiRNAs derived from the termini of RSV genomes

**Fig 1:**
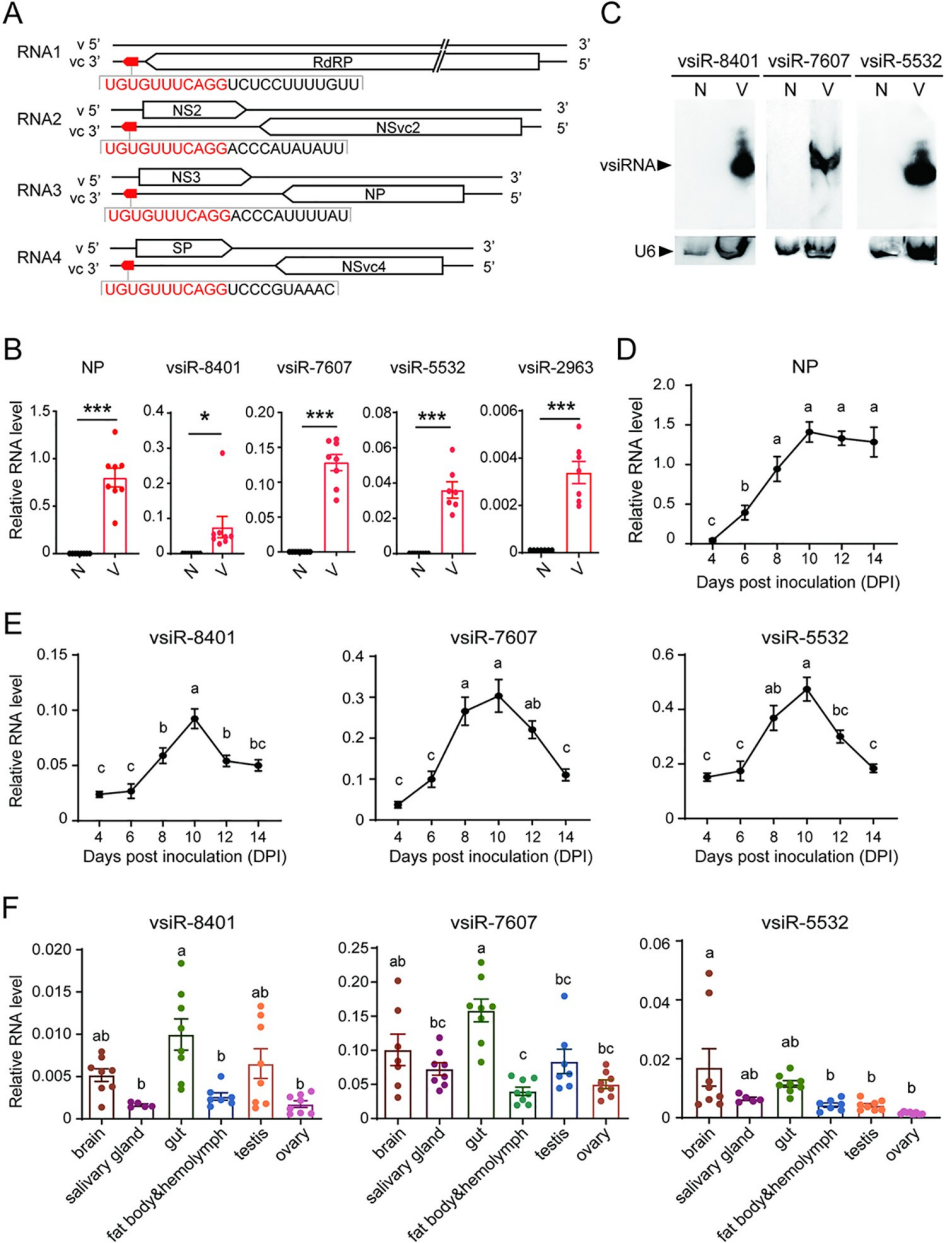
Characterization of vsiRNAs derived from the termini of RSV genomes. (A) Schematic representation of the four RSV-derived vsiRNAs. Four vsiRNAs are derived from the 3’ termini of viral complementary genomic RNAs (vcRNAs) of RSV. The red arrow represents the direction and location of vsiRNA sequences. A total of 11 nucleotides are shared by the four vsiRNAs and are marked in red. (B) The RNA levels of viral *NP* relative to that of *EF2* and the RNA levels of vsiRNAs relative to that of U6 snRNA in nonviruliferous (N) and viruliferous (V) third-instar planthoppers (n = 8). Values were compared by Student’s t test. *, *P* < 0.05. ***, *P* < 0.001. (C) Identification of vsiR-8401, vsiR-7607, and vsiR-5532 in nonviruliferous (N) and viruliferous (V) third-instar planthoppers by Northern blotting using biotin-labeled LNA oligonucleotide probes. (D) and (E) The RNA levels of *NP* relative to that of *EF2* (D) and the RNA levels of three vsiRNAs relative to that of U6 snRNA (E) in nonviruliferous planthoppers at different days post inoculation with RSV crude preparations (n = 8). (F) The RNA levels of three vsiRNAs relative to that of U6 snRNA in six organs of viruliferous planthopper adults (n = 8). From (D) to (F), different letters indicate significant differences in Tukey’s multiple comparison test. Graphs show mean values and standard errors.

Sequencing of small RNAs in our previous work showed that RSV generated numerous vsiRNAs during infection in small brown planthopper and rice [27]. Four vsiRNAs derived from the 3’-termini of viral complementary genomic RNAs (vcRNAs) were identified as vsiR-8401 (5’-UUGUUUUCCUCUGGACUUUGUGU-3’) with 23 nt from vcRNA1, vsiR-7607 (5’- UUAUAUACCCAGGACUUUGUGU-3’) with 22 nt from vcRNA2, vsiR-5532 (5’-UAUUUUACCCAGGACUUUGUGU-3’) with 22 nt from vcRNA3, and vsiR-2963 (5’- CAAAUGCCCUGGACUUUGUGU-3’) with 21 nt from vcRNA4. Their sequences shared 11 nt (Fig 1A). Sanger sequencing and expression quantification by quantitative real-time PCR (qPCR) in the viruliferous planthoppers confirmed their identity as vsiRNAs (Fig 1B). Northern blot assays showed a distinct band for vsiR-8401, vsiR-7607, and vsiR-5532 in viruliferous planthoppers (Fig 1C) but not for vsiR-2963, probably owing to the extremely low amount of vsiR-2963, as demonstrated in sRNA sequencing [27]; thus, vsiR-2963 was excluded from the subsequent studies.

When the nonviruliferous third-instar nymphs were inoculated with RSV crude preparations, the viral amount in terms of *NP* RNA level peaked at 10 d post inoculation (DPI) and maintained this level until 14 DPI (Fig 1D). The expression patterns of vsiR-8401, vsiR-7607, and vsiR-5532 were similar. More vsiR-8401, vsiR-7607, and vsiR-5532 were generated with viral replication, and their amounts were maximal at 8 or 10 DPI when viral replication peaked and then dropped down to a certain level (Fig 1E). In the adults of viruliferous planthoppers, the three vsiRNAs were ubiquitously distributed in various tissues, including the brain, salivary gland, gut, fat body and hemolymph, testis, and ovary (Fig 1F).

### Biogenesis of the three vsiRNAs is dependent on both Dicer1 and Dicer2

**Fig 2:**
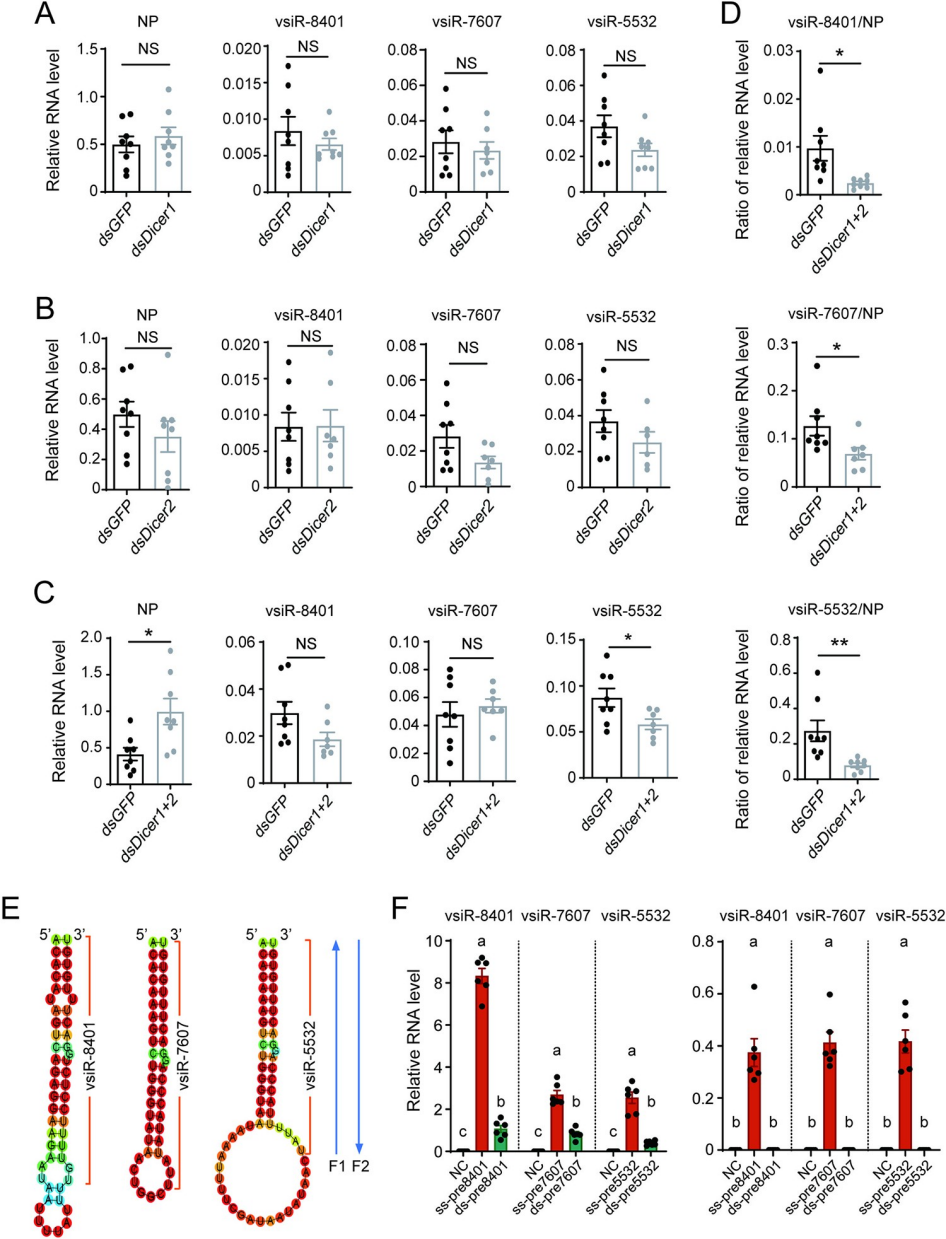
Biogenesis of the three vsiRNAs. (A)-(C) The RNA levels of viral *NP* relative to that of *EF2* and the RNA levels of three vsiRNAs relative to that of U6 snRNA in nonviruliferous planthoppers after injection of RSV crude preparations with ds*Dicer1*-RNA (A), or ds*Dicer2*-RNA (B), or both ds*Dicer1*- and ds*Dicer2*-RNA (C) for 6 d (n = 7 or 8). Injection of RSV crude preparations with ds*GFP*-RNA was used as control. (D) The ratios of relative RNA levels of three vsiRNAs to *NP* in the samples of (C). Values were compared by Student’s t test. *, *P* < 0.05. **, *P* < 0.01. NS, no significant difference. (E) Putative precursor sequences of single-stranded RNA with the panhandle structure for each vsiRNA. These precursor sequences are composed of 5’ and 3’ UTRs of viral RNA1, RNA2, or RNA3 segment. The vsiRNA sequences are indicated by orange brackets. Two forward primers F1 and F2 used in qPCR (F) are shown. (F) The RNA levels of the three vsiRNAs produced from single-stranded RNA precursors (ss-pre) and dsRNA precursors (ds-pre) relative to that of *Drosophila* U6 snRNA in S2 cells using primer F1 (left panel) and F2 (right panel) (n = 6). NC, blank cells as negative control. Different letters indicate significant differences by Tukey’s multiple comparison test.

To explore the biogenesis of the three vsiRNAs, *Dicer1* or *Dicer2* was silenced by injection of a mixture of double-stranded RNAs (dsRNA) for each gene and RSV crude preparations in nonviruliferous planthoppers. Compared to the negative control group, which was injected with ds*GFP*-RNA and RSV, silencing *Dicer1* or *Dicer2* did not affect the amounts of vsiR-8401, vsiR-7607 or vsiR-5532 or viral *NP* at 6 DPI (Figs 2A, 2B, S1A, and S1B). When the expression of *Dicer1* and *Dicer2* was knocked down simultaneously with injection of a dsRNA mixture for the two genes, the RNA level of viral *NP* was markedly increased, and only vsiR-5532 amount dropped significantly (Figs 2C and S1C). After excluding the influence of viral titer with normalization to *NP* level, the amounts of the three vsiRNAs were significantly reduced (Fig 2D). These results revealed that both Dicer1 and Dicer2 were required for the biogenesis of the three vsiRNAs in planthoppers.

### The three vsiRNAs probably are generated from viral terminal panhandle structure

The 5’ and 3’ UTR of each genomic RNA of RSV form a panhandle structure. To clarify whether the three vsiRNAs were generated from this featured structure or replication intermediates, we tried to identify the most likely vsiRNAs* (the complementary strands) for each vsiRNAs in the previously constructed sRNA libraries [27] to uncover whether they are perfect duplexes (replication intermediates as source) or duplexes carrying mismatches (panhandle structure as source). No vsiRNA* candidates derived from replication intermediates were found for the three vsiRNAs. Instead, a vsiR-5532* “5’-ACACAAAGTCTGGGTAATAAAATTTTC-3” and a vsiR-7607* “5’-ACACAAAGTCTGGGTATAACT-3” with 3 nt shorter than vsiR-7607 were retrieved from terminal panhandle structures. However, vsiR-8401* was not detected.

To further verify the source of the three vsiRNAs, two types of putative precursor sequences for each vsiRNA were synthesized. One was ssRNA with the panhandle structure formed by the 5’ and 3’ UTR of RSV RNA1, RNA2, or RNA3 segment (Fig 2E). The other was a perfect duplex of dsRNA to mimic replication intermediates of the three RNA termini. The lengths of these putative precursor sequences ranged from 44 nt to 60 nt. After these putative precursor sequences were transfected to *Drosophila* S2 cells for 24 h, each vsiRNA was amplified with two pairs of primers by qPCR for sequencing and quantification. Sequencing the products from the first pair of primers with forward primer F1 can confirm vsiRNA 3’ termini and sequencing the products from the second pair of primers with forward primer F2 can confirm vsiRNA 5’ termini (Fig 2E). Using the forward primer F1, both putative precursor sequences generated the three vsiRNAs with proper 3’ termini and the ssRNAs generated more vsiRNAs than the dsRNAs (Fig 2F). On the other hand, only the ssRNA precursors produced the three vsiRNAs with proper 5’ termini using the forward primer F2 while the 5’ termini of products from dsRNA precursors were random (Fig 2F). These results showed that the three vsiRNAs probably originated from viral terminal panhandle structure rather than replication intermediates.

### The three vsiRNAs facilitate RSV replication in planthoppers

**Fig 3:**
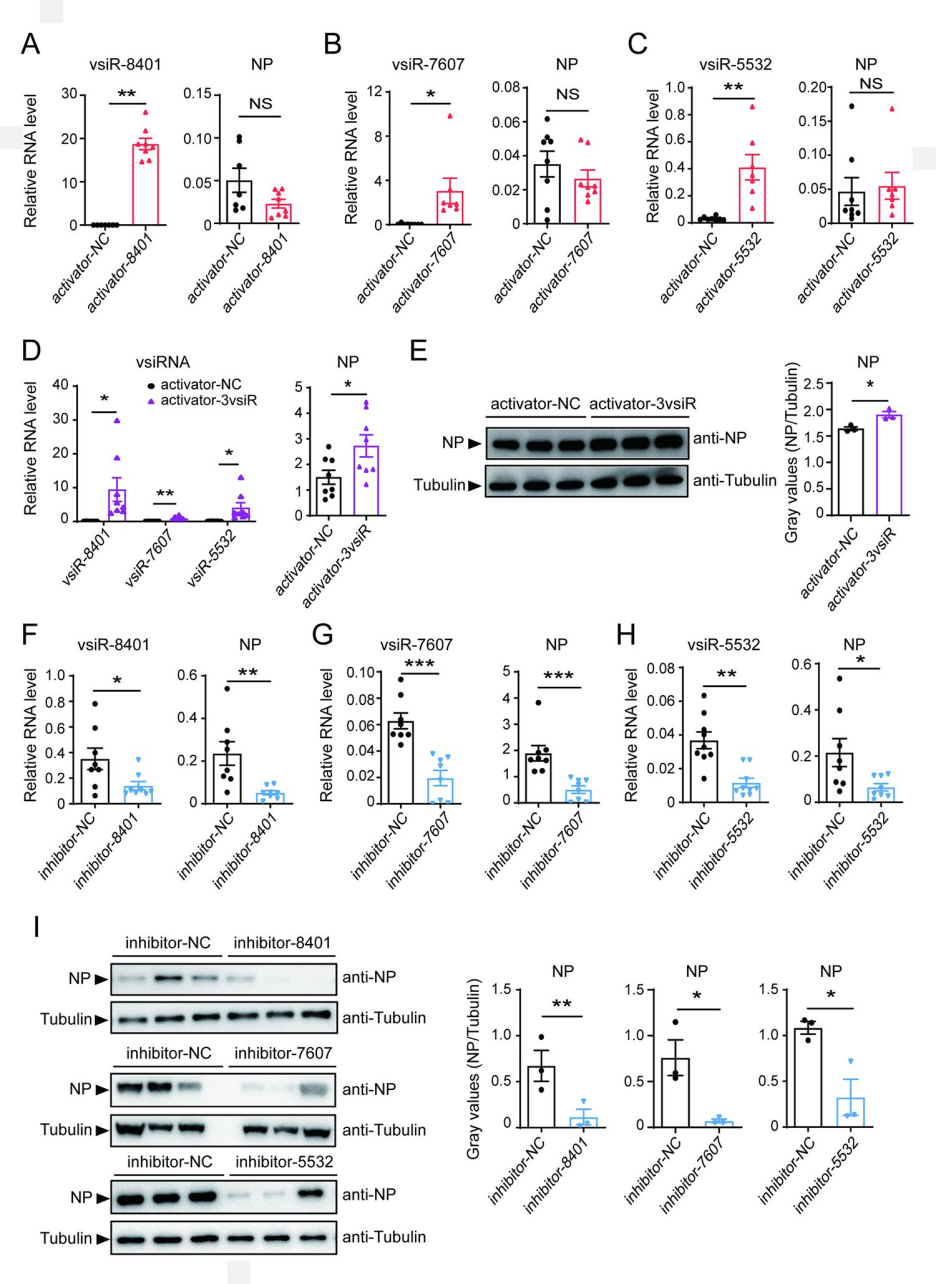
The three vsiRNAs facilitate RSV accumulation in planthoppers. (A)-(C) The RNA levels of vsiRNAs relative to that of U6 snRNA and the RNA levels of *NP* relative to that of *EF2* in nonviruliferous planthoppers after injection with a mixture of RSV crude preparations and activators of vsiR-8401 (A), vsiR-7607 (B), vsiR-5532 (C), or three vsiRNA (3vsiR) activators (D) for 6 d (n = 7 or 8). (E) Western blot showing the protein level of NP (n = 3) in the samples of (D) using anti-NP monoclonal antibody. (F)-(H) The RNA levels of vsiRNAs relative to that of U6 snRNA and the RNA levels of *NP* relative to that of *EF2* in nonviruliferous planthoppers after injection with a mixture of RSV and inhibitors of vsiR-8401 (F), vsiR-7607 (G), or vsiR-5532 (H) for 6 d (n = 8). (I) Western blot showing the protein level of NP (n = 3) in the samples of (F)-(H) using anti-NP monoclonal antibody. For (E) and (I), an anti-tubulin polyclonal antibody was used to measure tubulin as an internal control. Gray values show the relative optical densities of NP to that of tubulin. NC, negative control. Graphs show mean values and standard errors. Values were compared by Student’s t test. NS, no significant difference. *, *P* < 0.05. **, *P* < 0.01. ***, *P* < 0.001.

The roles of the three vsiRNAs in RSV replication were explored by injection of the synthetic activator or inhibitor of each vsiRNA and RSV crude preparations into nonviruliferous planthoppers. Compared to regular vsiRNA mimic, activator and inhibitor are chemically modified and more stable and functional *in vivo*. Inoculation of the synthetic activator for each vsiRNA did not influence viral amount in terms of *NP* RNA level at 6 d after RSV infection (Fig 3A–3C). When the mixture of the three vsiRNA activators were inoculated, *NP* RNA and protein levels significantly increased (Fig 3D and 3E). On the other hand, significant decreases in *NP* RNA and protein levels were observed when the inhibitor of each vsiRNA, i.e., the complementary sequence to the vsiRNA, was injected in comparison to the control inhibitor (NC) (Fig 3F–3I). Owing to the 11 nt identical sequences shared by the three vsiRNAs, we checked the possible interactive effects of each inhibitor on the expression of the other two vsiRNAs. The inhibitor of vsiR-7607 functioned specifically, not affecting the expression of the other two vsiRNAs (S2A Fig), while the vsiR-8401 inhibitor significantly downregulated the levels of both vsiR-7607 and vsiR-5532 (S2B Fig), and the vsiR-5532 inhibitor also reduced the expression of vsiR-7607 (S2C Fig). This demonstrated that the lack of the three vsiRNAs, or at least vsiR-7607, was detrimental to RSV accumulation and that the amounts of the three vsiRNAs maintained endogenously were sufficient to support viral infection.

### vsiR-8401 and vsiR-5532 target viral RNAs in a manner similar to miRNA, but result in an opposite effect on viral accumulation

**Fig 4:**
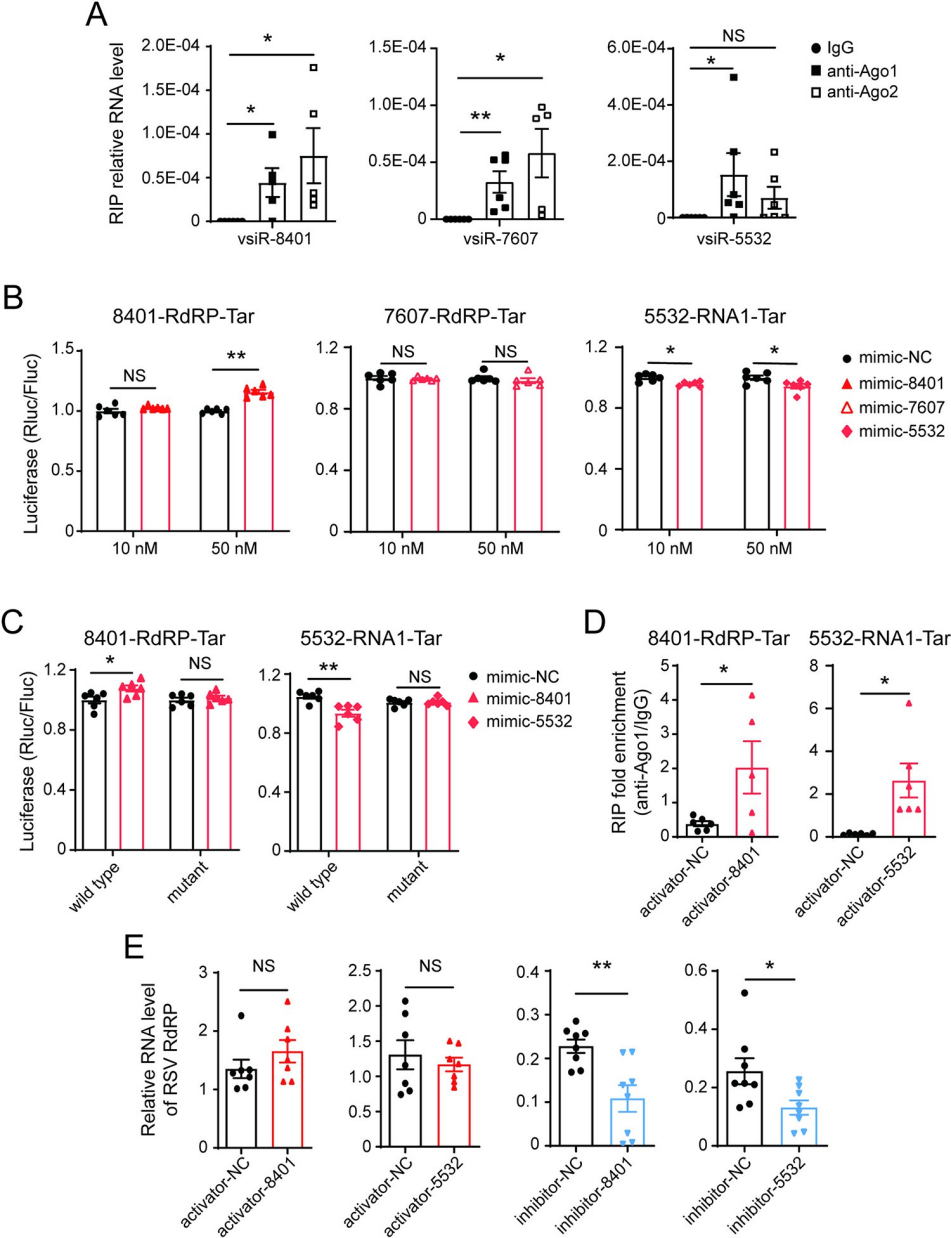
vsiR-8401 and vsiR-5532 target viral RNAs in a miRNA-like manner. (A) The RNA levels of vsiR-8401, vsiR-7607, and vsiR-5532 in the immunoprecipitated fraction relative to those in the input from viruliferous planthoppers, measured by RIP combined with qPCR (n = 5 or 6). Mouse IgG was used as a negative control. (B) Dual luciferase reporter assays in *Drosophila* S2 cells cotransfected with recombinant psiCHECK2 plasmids containing the predicted target (Tar) sequence of the three vsiRNAs and vsiRNA mimic (n = 6). (C) Dual luciferase reporter assays in S2 cells cotransfected with recombinant psiCHECK2 plasmids containing the wild-type and the mutant target (Tar) of vsiR-8401 or vsiR-5532 and 50 nM of vsiR-8401 or vsiR-5532 mimic (n = 6). The activity of *Renilla* luciferase (Rluc) relative to that of firefly luciferase (Fluc) is presented. (D) Relative enrichment of the targets (Tar) of vsiR-8401 and vsiR-5532 in nonviruliferous planthoppers measured by RIP combined with qPCR after injection with activators of vsiR-8401 or vsiR-5532 for 6 d (n = 5-6). Mouse IgG instead of the anti-Ago1 monoclonal antibody was used as a negative control. (E) The RNA level of *RdRP* relative to that of *EF2* in nonviruliferous planthoppers after injection with a mixture of RSV crude preparations and activators or inhibitors of vsiR-8401 and vsiR-5532. NC, negative control. Values were compared by Student’s t test. NS, no significant difference. *, *P* < 0.05. **, *P* < 0.01.

vsiRNAs usually activate RNA silencing to suppress virus infection by guiding RISC to degrade viral genomes [11]. This canonical function of vsiRNAs did not explain the positive role of the three vsiRNAs in RSV infection. Whether the three vsiRNAs work on viral RNAs in a miRNA-like manner was explored. Ago1 and Ago2 proteins recruit miRNAs and siRNAs, respectively, in RISCs. First, we performed RNA immunoprecipitation combined with qPCR (RIP-qPCR) in viruliferous planthoppers using homemade anti-Ago1 or anti-Ago2 monoclonal antibodies [28]. vsiR-8401 and vsiR-7607 were enriched in both Ago-immunoprecipitated complexes, and vsiR-5532 was only enriched in Ago1-immunoprecipitated complexes in comparison to the IgG immunoprecipitation control (Fig 4A), indicating that the three vsiRNAs were involved in the miRNA pathway.

Second, the candidate targets within viral genomic RNAs or vcRNAs were predicted using the miRanda [29] and RNAhybrid [30] algorithms. Both algorithms indicated that the coding region of *RdRP* on vcRNA1 could be targeted by vsiR-8401 at the site from 7698 to 7720 nt and by vsiR-7607 at the site from 3215-3235 nt but not by vsiR-5532 (S1 Table). When only using RNAhybrid to perform calculations for vsiR-5532 with a cutoff threshold of -18 kcal mol^−1^, viral RNA1 was shown to be targeted by vsiR-5532 at the site from 1866 to 1897 nt, which is a region that has reverse complementarity to *RdRP*, in addition to the other four possible targets (S1 Table).

Then, the direct interaction between vsiRNAs and their binding sites on vcRNA1 or RNA1 was further verified through dual-luciferase assays in S2 cells using regular vsiRNA mimic. The luciferase activity of cells transfected with the construct containing the vcRNA1 *RdRP* target site and 50 nM vsiR-8401 mimic was 1.2-fold of that in the control group, while the luciferase activities of cells transfected with the construct containing the RNA1 target site decreased by around 5% in the presence of 10 and 50 nM vsiR-5532 mimic (Fig 4B). Mutations at the seed region of the binding sites abolished these effects of vsiR-8401 and vsiR-5532 mimic (Fig 4C). The vsiR-7607 mimic did not affect the luciferase activities of cells transfected with the construct containing the vcRNA1 *RdRP* target (Fig 4B), indicating that vsiR-7607 did not bind vcRNA1 *RdRP*. RIP-qPCR assays of viruliferous planthoppers showed that the target sequences of vsiR-8401 and vsiR-5532 on vcRNA1 and RNA1 were enriched in the Ago1-immunoprecipitated complexes after injection of vsiRNA activators compared to injection of a control activator (NC) (Fig 4D). Inoculation of the inhibitor for vsiR-8401 or vsiR-5532 decreased the RNA levels of *RdRP*, while the activator for each vsiRNA did not affect *RdRP* levels at 6 d after RSV infection (Fig 4E). These results demonstrate that vsiR-8401 and vsiR-5532 target viral *RdRP* and genomic RNA1 in a miRNA-like manner, but their effects on these viral RNAs seem not always consistent. Such direct interactions of the three vsiRNAs with viral RNAs may not be the main force to promote RSV accumulation.

### *DOPA decarboxylase* is the common target of the three vsiRNAs in planthoppers

**Fig 5:**
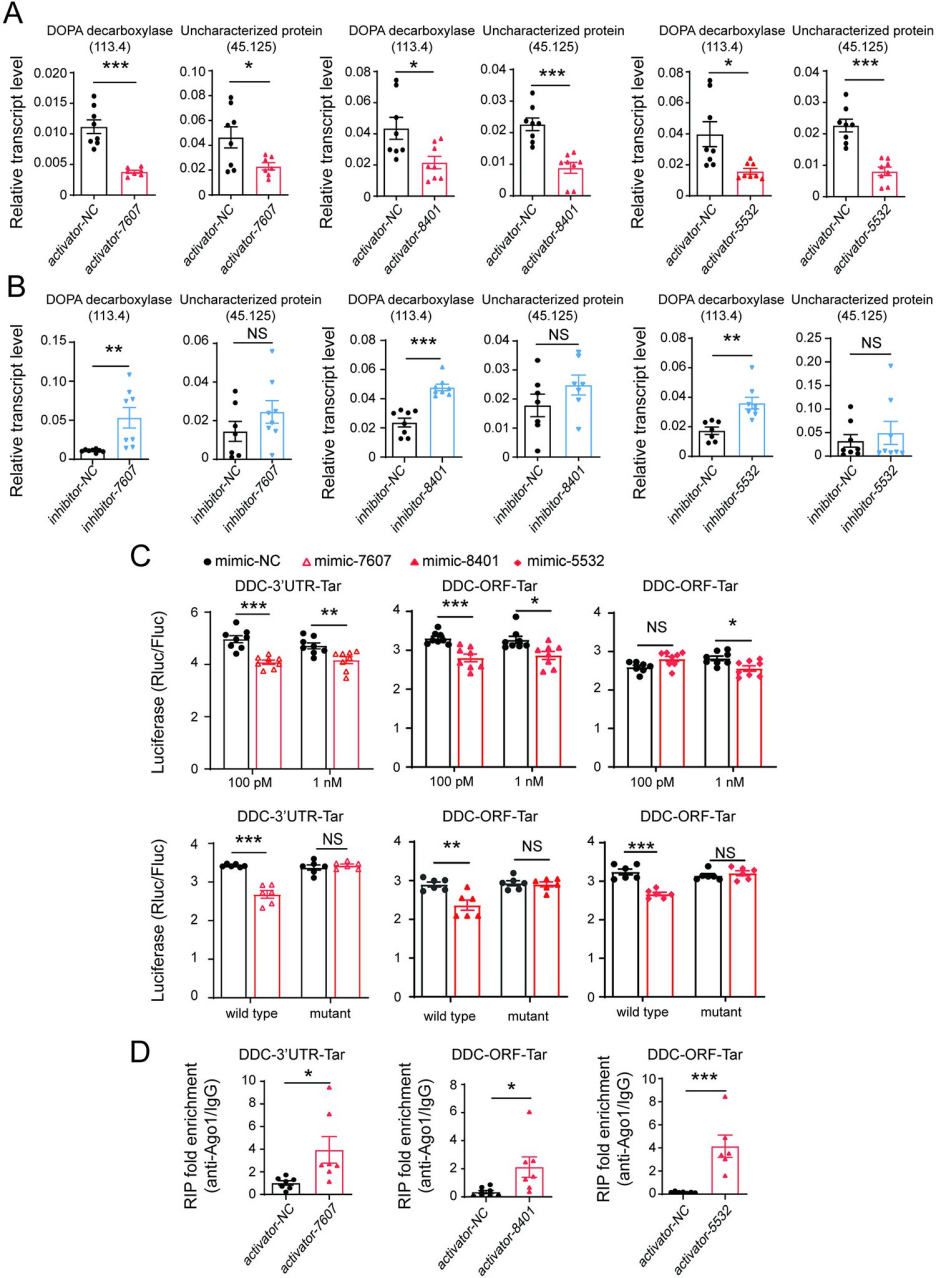
DOPA decarboxylase is the common target of the three vsiRNAs in planthoppers. (A) The transcript levels of *DOPA decarboxylase* (evm.model.Contig113.4) and *uncharacterized protein* (evm.model.Contig45.125) relative to that of *EF2* in the nonviruliferous planthoppers after injection with activator of vsiR-7607, vsiR-8401, or vsiR-5532 for 3 d (n = 7 or 8). (B) The transcript levels of *DOPA decarboxylase* and *uncharacterized protein* relative to that of *EF2* in the nonviruliferous planthoppers after injection with the mixture of RSV and the inhibitor of each vsiRNA for 6 d (n = 7 or 8). (C) Dual luciferase reporter assays in S2 cells cotransfected with recombinant psiCHECK2 plasmids containing the predicted *DOPA decarboxylase* (*DDC*) target (Tar) sequence of each vsiRNA or their mutants and vsiRNA mimic (n = 6 or 8). For the mutant measurement, 1 nM vsiR-7607, 100 pM vsiR-8401, or 1 nM vsiR-5532 mimic was applied. The activity of *Renilla* luciferase (Rluc) relative to that of firefly luciferase (Fluc) is presented. (D) Relative enrichment of *DDC* targets (Tar) of the three vsiRNAs in the nonviruliferous planthoppers measured by RIP combined with qPCR after injection with an activator of each vsiRNA for 3 d (n = 5 or 6). Mouse IgG instead of the anti-Ago1 monoclonal antibody was used as a negative control. NC, negative control. Values were compared by Student’s t test. NS, no significant difference. *, *P* < 0.05. **, *P* < 0.01. ***, *P* < 0.001.

In addition to directly targeting RSV, the three vsiRNAs probably regulate the gene expression of insect vectors to enhance virus infection, especially vsiR-7607, which does not seem to target the virus directly. To identify the planthopper genes regulated commonly by the three vsiRNAs, transcriptomic analyses were conducted on nonviruliferous planthoppers after treatment with 12.5 μM vsiR-7607 activator or NC activator for 3 d as the first step of screening. At least 49 million clean reads were obtained, and the Q30 value was higher than 91% for each sample (S2 Table). Compared to the NC group, 175 genes were downregulated and 37 genes were upregulated at least 2-fold (*P* < 0.05) by the vsiR-7607 activator (S3 and S4 Tables). Among the 212 differentially expressed genes (DEGs), two genes were potential targets of vsiR-7607, as predicted by both the RNAhybrid and miRanda algorithms, but they were not targets of vsiR-8401 or vsiR-5532 (S5 Table). When only using RNAhybrid to predict targets with a cutoff threshold of -18 kcal mol^−1^, 26 DEGs were screened as common targets of the three vsiRNAs. Among the 26 genes, 19 were downregulated and 7 were upregulated upon treatment with the vsiR-7607 activator in the transcriptomic analyses (S5 Table). qPCR showed that 12 of the 26 DEGs were consistent with the transcriptomic analyses and were downregulated by the vsiR-7607 activator (Figs 5A and S3). In the nonviruliferous planthoppers that were injected with activators of vsiR-8401 or vsiR-5532, only 2 of the 12 DEGs were also downregulated (Figs 5A and S4). These two genes putatively encoded DOPA decarboxylase (DDC, evm.model.Contig113.4) in the gene set of small brown planthopper) and an uncharacterized protein (evm.model.Contig45.125). When the inhibitors of vsiR-7607, vsiR-8401, or vsiR-5532 were injected with RSV crude preparations into nonviruliferous planthoppers for 6 d, the transcript level of *DDC* significantly increased with the treatment of each vsiRNA inhibitor, while the transcript level of evm.model.Contig45.125 did not change (Fig 5B). Therefore, *DDC* would be the best candidate target gene of the three vsiRNAs.

The open reading frame (ORF) of *DDC* was 1434 bp. The predicted target sites were from 1442 to 1464 bp downstream of the ORF in the 3’ UTR for vsiR-7607, from 45 to 71 bp in the ORF for vsiR-8401, and from 26 to 48 bp in the ORF for vsiR-5532. The interactions between the three vsiRNAs and their target sites in *DDC* were verified using dual-luciferase assays in S2 cells. The luciferase activities of cells transfected with the construct containing putative target sites significantly decreased by 5% to 10% in the presence of the corresponding vsiRNA mimic of at least one concentration compared to the control group (Fig 5C). Mutations of the target sites at the seed region abolished the inhibition of luciferase activities by the corresponding vsiRNA mimic (Fig 5C). RIP-qPCR showed that the three target sequences in *DDC* were enriched in the Ago1-immunoprecipitated complexes from viruliferous planthoppers after injection of the activator of vsiR-7607, vsiR-8401, or vsiR-5532 compared to the injection of a control activator (NC) (Fig 5D). To verify the cleavage of *DDC* by the three vsiRNAs, 5’ RNA Ligase Mediated Rapid Amplification of cDNA Ends (5’RLM-RACE) was performed in viruliferous planthoppers. For vsiR-8401 and vsiR-5532, which bind to the site from 26 to 71 bp in the ORF of *DDC*, we successfully obtained and sequenced 8 clones. The 5’ ends of these clones were located approximately 200 or 400 bp downstream of the binding sites (S5 Fig). For vsiR-7607, 4 clones were sequenced and the 5’ ends of these clones were located approximately 200 bp downstream of the binding site (S5 Fig). These results demonstrate that *DDC* is a common target gene of the three vsiRNAs and that vsiRNA-mediated *DDC* degradation follows the classical 5’-to-3’ mRNA degradation pathway in planthoppers.

### DOPA decarboxylase participates in the PPO-mediated antiviral immune reaction

**Fig 6:**
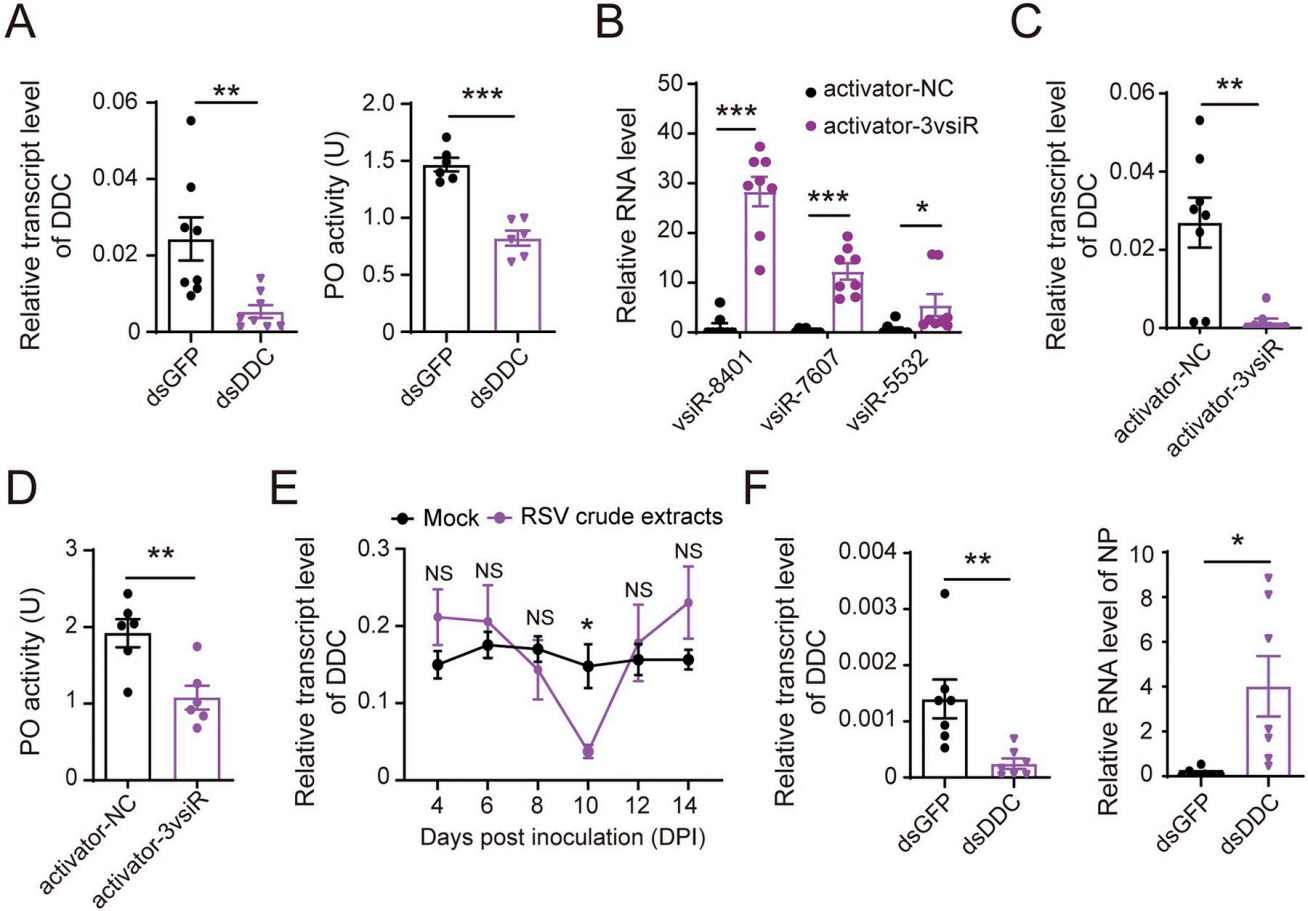
DOPA decarboxylase (DDC) participates in the PPO-mediated antiviral immune reaction. (A) The transcript levels of *DDC* relative to that of *EF2* (n = 7 or 8) and PO activity (n = 6) in the whole body of nonviruliferous planthoppers after injection with ds*DDC*-RNA or ds*GFP*-RNA for 3 d. (B) and (C) The RNA levels of the three vsiRNAs relative to that of U6 snRNA (B) and the transcript levels of *DDC* relative to that of *EF2* (C) in the nonviruliferous planthoppers after injection with the mixture of activators of the three vsiRNAs (3vsiR) for 3 d (n = 7 or 8). (D) PO activity in the whole body of nonviruliferous planthoppers after injection with the mixture of activators of the three vsiRNAs (3vsiR) for 3 d (n = 6). NC, negative control. (E) The transcript levels of *DDC* relative to that of *EF2* in nonviruliferous planthoppers at different days post inoculation with RSV crude extracts or the extracts from nonviruliferous planthoppers (mock) (n = 8). (F) The transcript levels of *DDC* and the RNA levels of *NP* relative to that of *EF2* in nonviruliferous planthoppers after injection with a mixture of RSV crude preparations and ds*DDC*-RNA or ds*GFP*-RNA for 6 d (n = 7 or 8). Values were compared by Student’s t test. NS, no significant difference. *, *P* < 0.05. **, *P* < 0.01. ***, *P* < 0.001.

One of the functions of DDC is to catalyze the conversion of L-Dopa to dopamine, which serves as a substrate to generate melanin in the proteolytic prophenoloxidase (PPO) pathway, restricting or killing pathogens [31]. Our previous work revealed the antiviral immune role of the PPO pathway toward RSV and demonstrated that RSV suppressed phenoloxidase (PO) activity during infection in planthoppers [32]. To clarify the function of the target DDC in the PPO pathway, PO activity was measured in nonviruliferous planthoppers after injection of ds*DDC*-RNA for 3 d. Knockdown of *DDC* expression reduced PO activity by 45.1% (Fig 6A), proving that the target DDC positively regulates the PPO immune reaction. After the three vsiRNA activators were injected into nonviruliferous planthoppers for 3 d, the transcript level of *DDC* was downregulated (Fig 6B and 6C), and the PO activity dropped accordingly (Fig 6D). With the infection of RSV in planthoppers, the transcript level of *DDC* dropped at 10 DPI when the amounts of the three vsiRNAs were maximal (Figs 1E and 6E). When ds*DDC*-RNA and RSV crude preparations were injected into nonviruliferous planthoppers, the expression of *DDC* was knocked down, and the viral amount in terms of *NP* RNA level was dramatically enhanced at 6 DPI (Fig 6F). These results indicate that the target DDC participates in the PPO-mediated antiviral immune reaction.

### The three vsiRNAs facilitate RSV replication in rice

**Fig 7:**
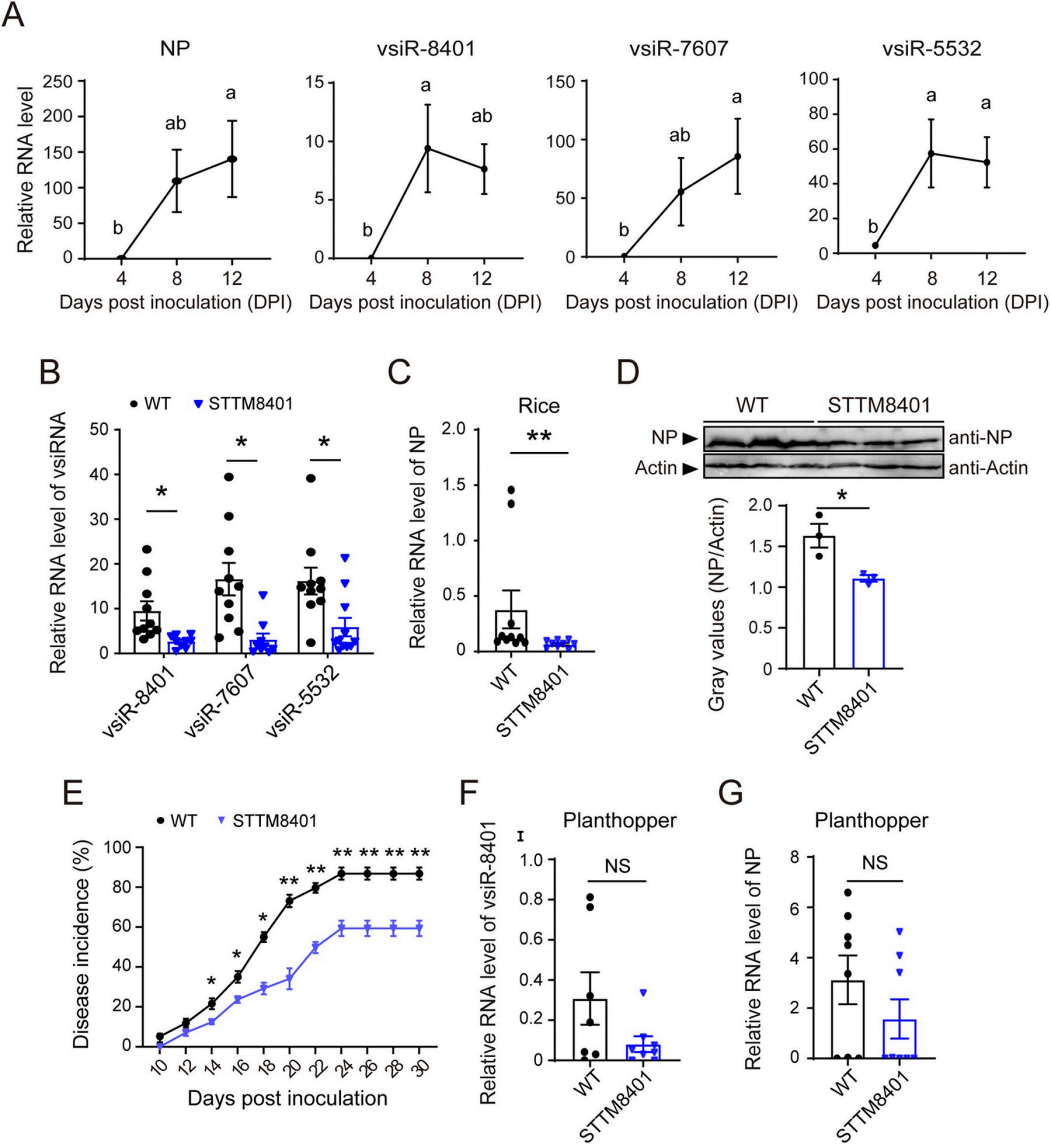
The three vsiRNAs facilitate RSV replication in rice. (A) The RNA levels of *NP* relative to that of rice *UBQ10* and the three vsiRNAs relative to that of rice U6 snRNA in rice leaves at different days post inoculation with viruliferous planthoppers (n = 8). Different letters indicate significant differences in Tukey’s multiple comparison test. (B) and (C) Relative RNA levels of the three vsiRNAs and *NP* in the T2 generation of vsiR-8401-silenced lines (STTM8401) compared to the wild-type (WT) rice post inoculation with viruliferous planthoppers for 7 d. Graphs show mean values and standard errors. (D) Western blot showing NP protein levels (n = 3) in the samples of (C) using anti-NP monoclonal antibody. An anti-actin polyclonal antibody was used to measure actin as an internal control. Gray values show the relative optical densities of NP to that of actin. (E) The disease incidence of WT and STTM8401 rice fed on by viruliferous planthoppers for 7 d. Five rice seedlings per replicate and six replicates were applied. (F-G) Relative RNA levels of the three vsiR-8401 (F) and *NP* in viruliferous planthoppers that consistently fed on WT and STTM8401 rice for 7 d (n = 7 to 8). From (B) to (G), values were compared by Student’s t test. NS, no significant difference. *, *P* < 0.05. **, *P* < 0.01.

In contrast with the positive roles in insect vectors, whether the three vsiRNAs exerted comparable functions to RSV replication in rice was further explored. vsiR-8401, vsiR-7607 and vsiR-5532 were detected in RSV-infected rice, and their amounts continually increased with viral replication and maintained high levels within 12 d (Fig 7A). Considering that the vsiR-8401 inhibitor also reduced the abundancy of vsiR-7607 and vsiR-5532 in planthoppers (S2B Fig), we created vsiR-8401 knockdown rice lines (STTM8401) using short tandem target mimic (STTM) technique. The transgenic lines exhibited comparable plant height, 1000-grain weight, grain width and length to those of the wild-type (WT) rice (S6 Fig). The T2 generation of transgenic rice fed viruliferous planthoppers for 7 d to be inoculated with RSV. As expected, the amount of vsiR-8401, as well as vsiR-7607 and vsiR-5532, was much lower in STTM8401 lines compared to that in the WT rice at 7 dpi (Fig 7B). A significant decrease of viral titer in terms of RSV *NP* RNA and protein level was observed in STTM8401 lines (Fig 7C and 7D), indicating that the three vsiRNAs promoted RSV replication in rice. Moreover, the disease incidence of STTM8401 was also lower than the WT under greenhouse conditions, with only 59.3% in STTM8401 line versus 86.8% in the WT within 30 dpi (Fig 7E). On the other hand, we also examined the vsiR-8401 levels and viral loads in viruliferous planthoppers fed the transgenic rice for 7 d. Insects consuming STTM8401 lines showed a trend towards lower vsiR-8401 and *NP* RNA levels versus WT-fed insects, albeit without statistically significant differences. While a direct negative effect of STTM8401 on the virus cannot be completely ruled out, it is unlikely due to the conformation of the viral genome/replication intermediates in the region where the vsiRNAs originate. These results indicated a possible positive role of the three vsiRNAs in RSV replication in rice.

## Discussion

**Fig 8:**
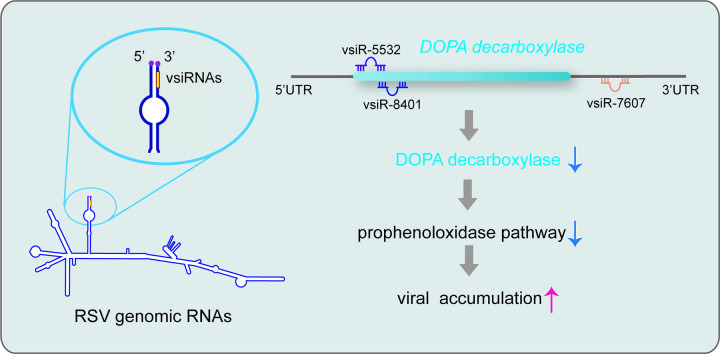
Model of promoting viral accumulation in insect vectors by RSV vsiRNAs generated from the conserved terminal panhandle structure.

The RNAi system is well known to modulate antiviral defense during viral infection in animals and plants. In this study, we revealed an unexpected phenomenon of vsiRNAs positively regulating viral infection in insect vectors and host plants. These vsiRNAs were generated from the conserved terminal panhandle structure of a segmented, negative-strand RNA virus. They not only suppressed the antiviral immune reaction in insect vectors by coordinately downregulating *DDC* expression in the PPO pathway (Fig 8), but also promoted viral replication in host plants. This study elucidated a unique function of vsiRNAs opposite to their usual antiviral effects on viral infection, and shed light on developing resistant rice resources to combat viral diseases in future.

Many arboviruses that infect vertebrates and plants depend on insect vectors for transmission and frequently cause epidemics in large areas [33,34]. A delicate balance between antiviral immune responses and viral replication has evolved in insect vectors to keep viral load at a nonpathogenic level and at the same time ensure transmission competence. However, even though virus-derived small RNAs function in the most important antiviral innate immune pathways in insects, their functions have seldom been reported. DENV-vsRNA-5, a vmiRNA derived from the 3’ stem–loop of the DENV-2 genome, suppresses viral replication by targeting viral nonstructural protein 1 in mosquito cells [17]. KUN-miR-1, a vmiRNA generated from the 3’ stem–loop of the flavivirus West Nile virus genome, benefits viral replication by upregulating mosquito *GATA4* expression [16]. Our group reported that vsiR-3397 from RSV RNA3 produced an antiviral effect in planthoppers by inhibiting insect miR-263a expression after directly binding to the promoter region of miR-263a [35]. In this study, we found that three vsiRNAs from the genomic termini of RSV promoted viral replication by suppressing planthopper *DDC* expression. These diverse functions of vsiRNAs and vmiRNAs are achieved in a miRNA-like manner, i.e., partial sequence complementarity with target genes. The functional diversity of virus-derived small RNAs may help arboviruses adapt to insect vectors easily by flexibly regulating viral load.

The synthesis and functional pathways of the three vsiRNAs are complicated. vmiRNAs usually possess ∼70 nt-long precursors with a conserved stem–loop structure and are easily detected in northern blots [16,17,36]. The three vsiRNAs of RSV do not possess a classical stem–loop precursor structure. Northern blot analysis did not reveal the longer precursors. Dicer1 is normally required for miRNA biogenesis, and Dicer2 mainly participates in siRNA biogenesis [37]. However, the biogenesis of the three RSV vsiRNAs depended on both Dicer1 and Dicer2. Furthermore, vsiR-8401 and vsiR-7607 were found to bind Ago1 and Ago2, indicating that they probably function in both miRNA and siRNA pathways. Usually vsiRNAs trigger an Ago-dependent RNAi pathway to specifically target and degrade viral RNAs [38]. However, the viral sequences that are fully complementary to the three vsiRNAs often form a panhandle structure during viral replication and translation, reducing the likelihood that these sequences will be bound and degraded by the three vsiRNAs. On the other hand, these vsiRNAs have more opportunities to act on insect genes in a miRNA-like manner. Interestingly, the three vsiRNAs targeted different regions of *DDC*. It is well known that miRNAs are involved in a sophisticated gene regulatory network. A miRNA can modulate multiple genes by targeting transcription factors, and in turn, one common gene can also be targeted by a cluster of miRNAs [39]. For example, endogenous miR-101, miR-129-5p and miR-221 target the 3’ UTR of *fragile X mental retardation gene 1* to cooperatively modulate its expression in human cells [40]. The human *cyclin-dependent kinase inhibitor 1A* gene is directly targeted by nearly 28 miRNAs, leading to gene expression inhibition [41]. In this study, we first revealed that multiple vsiRNAs coordinately regulate the expression of one common gene by binding to different regions of the gene.

Our work presents a case for the origination of vsiRNAs from the conserved terminal panhandle structure of negative-strand RNA viruses. It is widely accepted that vsiRNAs are produced from the dsRNA replication intermediates or from self-complementary dsRNA regions within the viral genome [11, 42]. However, the experimental evidence supporting the existence of long viral dsRNA intermediates and their susceptibility to DICER cleavage is lacking. Several negative-strand RNA viruses and positive-strand RNA viruses have been found to produce miRNA-like RNAs or siRNAs from specific structural features within their genomes. For example, miR-HA-3p, miR-VP-3p, and miR-nsp3-3p are processed from the stem-loop structure within gene coding regions in H5N1 influenza virus, Ebola virus and SARS-CoV-2, respectively [15,43,44], whereas KUN-miR-1 and DENV-vsiRNA-5 are derived from the stem-loop structure in viral genomic 3’ UTR of West Nile virus and Dengue virus [16,17]. In our study, we found that the three vsiRNAs of RSV are generated from the panhandle structure, which is formed by both the 5’ and 3’ UTRs of viral single-stranded genomes, instead of the replicative intermediates. This mechanism may be prevailing in negative-strand RNA viruses.

The three vsiRNAs may target different pathways in insect vectors and host plants. In insect vectors, we found that the three vsiRNAs directly regulated the PPO pathway by binding to the *DDC* gene. The PPO-mediated melanization reaction is one of the major innate immune pathways in insect hemolymph [45]. L-DOPA is converted to dopamine by DDC in nervous, epidermal and serotonergic cells [46]. PO oxidizes dopamine to produce quinones that are non-enzymatically polymerized to form dopamine melanin [47]. We previously found that the nonstructural protein NS3 of RSV impeded PO production from PPOs by occupying the proteolytic cleavage sites of PPOs [32]. Such multifaceted manipulation reflects the significance of the PPO pathway in the transmission of arboviruses. Once the PPO cascade was activated, distinct melanization appeared around RSV particles and seriously damaged viral stability in the hemolymph [32]. Plants do not have the PPO pathway. Instead, they have polyphenol oxidase (also known as tyrosinase), which converts L-DOPA to DOPA-quinone [48]. Plants utilize the oxidation of phenols catalyzed by polyphenol oxidase as a defense mechanism against bacterial pathogens such as *Pseudomonas syringae* [49]. Bioinformatics prediction denies the possibility of six polyphenol oxidases (LOC_Os01g58070, Os01g58100, Os04g53250, Os04g53260, Os04g53290, Os04g53300 in Rice Genome Annotation Project Database) as the targets of the three vsiRNAs in rice. We searched the homologous proteins of planthopper DDC in rice and found that the most similar was the tryptophan decarboxylase 2 (TDC2) (Genbank number NM_001422121.1) with 44.2% identity to planthopper DDC. Interestingly, bioinformatics prediction indicated that both vsiR-8401 and vsiR-5532 targeted the encoding region of *TDC2*, potentially downregulating *TDC2* expression. TDCs are a group of enzymes that convert tryptophan to tryptamine, which is hydroxylated to form serotonin by tryptamine 5-hydroxylase [50]. Serotonin plays a role in defending against pathogens in rice. When rice is infected by *Bipolaris oryzae*, serotonin content in rice leaves increases, helping establish effective physical defenses [51]. We postulate that two of the three vsiRNAs may interrupt the tryptophan pathway by targeting the *TDC2* gene to reduce the resistance of rice to RSV.

In summary, our work presents a novel finding of virus-derived small interfering RNAs in promoting arbovirus infection to insect vectors and host plants. This special positive regulation of vsiRNAs may be a conserved characteristic for vsiRNAs originating from terminal panhandle structure of segmented, negative-strand RNA viruses.

## Materials and methods

### Small brown planthopper strains

The nonviruliferous and viruliferous small brown planthopper strains used in this work were reared separately in glass incubators and screened using dot enzyme-linked immunosorbent assay (dot-ELISA) with a homemade monoclonal anti-NP antibody every three months as described previously [52].

### RNA extraction and cDNA synthesis

Total RNA was extracted with TRIzol reagent (Invitrogen, Carlsbad, CA, USA; 15596026) from 5-8 whole bodies of nonviruliferous third-instar nymphs after injection with RSV crude preparations; from 30 guts, 30 ovaries, 30 testes, 30 fat bodies, 30 salivary glands, 30 hemolymph, and 50 brains of viruliferous planthopper adults; from viruliferous third-instar nymphs after injection with vsiRNA activators or vsiRNA inhibitors; from nonviruliferous third-instar nymphs after injection with a mixture of RSV crude preparations and vsiRNA activators or vsiRNA inhibitors; and from one rice leaf. The quality of RNA was detected by a NanoDrop One (Thermo Scientific, Waltham, MA; 840-317400) and gel electrophoresis. Two microgram of RNA was reverse transcribed to cDNA using random primers (Promega, Madison, WI, USA; PAC1181) and an M-MLV reverse transcription system (Promega; PAM1701) following the manufacturer’s instructions. For vsiRNAs, 2 μg of RNA was reverse transcribed using the miRcute Plus miRNA First-Strand cDNA Kit (Tiangen, Beijing, China; 4992909).

### qPCR

qPCR was performed on a LightCycler 480 instrument II (Roche, Basel, Switzerland) using a LightCycler 480 SYBR Green I Master (Roche; 04887352001) or a miRcute miRNA qPCR Detection Kit (Tiangen; FP411). Planthopper *EF2* was used as an endogenous control for cellular mRNAs and viral RNAs, and planthopper U6 snRNA was used as an endogenous control for vsiRNAs. Six to eight replicates were prepared. The primers used in this work are listed in S6 Table. All PCR products were sequenced for validation.

### RIP-qPCR

RIP analysis was performed using a RIP-Kit (BersinBio, Guangzhou, China) based on our previous work [21]. Homemade anti-Ago1 and anti-Ago2 monoclonal antibodies [28] or normal mouse IgG (Abcam, Cambridge, UK; ab6708) were incubated with lysates from viruliferous fourth-instar planthopper nymphs for immunoprecipitation. One-tenth of the lysate supernatant served as the “Input” sample for reference. qPCR analysis was appropriately conducted to evaluate the levels of target RNAs in the immunoprecipitated (IP) fraction relative to the Input. For vsiRNA-target validation, anti-Ago1 monoclonal antibodies and IgG were incubated with extracts from nonviruliferous third-instar nymphs that were injected with activators of vsiR-8401, vsiR-7607, or vsiR-5532 for 3 d or 6 d for immunoprecipitation. Enriched RNA was extracted using TRIzol reagent and reverse transcribed into cDNA. qPCR was then performed to detect the transcript levels of target RNA sequences or the RNA levels of vsiRNAs. The RNA level of each target RNA relative to that in the IgG control sample is reported as the mean ± SE.

### Western blot assay

Total protein from planthoppers or rice leaves was extracted using 1 × PBS lysis buffer (pH7.3) (Beyotime Biotechnology, Shanghai, China; ST477) and separated by sodium dodecyl sulfate–polyacrylamide gel electrophoresis. Anti-β-tubulin polyclonal antibody (Abcam; ab15568), anti-plant Actin polyclonal antibody (EASYBIO, Beijing, China; BE0028), and homemade anti-NP monoclonal antibody [52] were used as primary antibodies. Immunoblot signals were detected using SuperSignal West Femto (Thermo Scientific; 34096). Three replicates were prepared. The density of proteins was quantified with the image analysis software ImageJ and normalized to that of tubulin or actin.

### Northern blot assay

Total RNA extracted from nonviruliferous and viruliferous planthoppers was used for northern blot analysis as described previously.^14^ Briefly, RNA was separated by 15% (wt/vol) denaturing polyacrylamide gels and electroblotted onto positively charged nylon membranes (Invitrogen; AM10102). Biotin-labeled LNA oligonucleotide probes (GenePharma, Shanghai, China) were generated for the antisense sequences of vsiR-8401 (5’-UUGUUUUCCUCUGGACUUUGUGU-3’), vsiR-7607 (5’- UUAUAUACCCAGGACUUUGUGU-3’), vsiR-5532 (5’-UAUUUUACCCAGGACUUUGUGU-3’), and U6 snRNA (5’-GGAACGAUACAGAGAAGAUUAG-3’). Probe hybridizations were performed at 37°C. Detection was carried out using SuperSignal West Femto.

### Double-stranded RNA synthesis and delivery

The dsRNAs were synthesized using the T7 RiboMAX Express RNAi System (Promega; P1700) following the manufacturer’s protocol. dsRNAs for *Dicer1*, *Dicer2*, *DDC*, and the green fluorescent protein gene (GFP) were generated by the corresponding primer pairs supplied in S6 Table. A total of 23 nL of dsRNAs for *Dicer1*, *Dicer2*, or *GFP* (12 μg μL^-1^) plus an equal volume of RSV crude extracts from viruliferous planthoppers [53] was delivered into nonviruliferous third-instar nymphs by microinjection using a Nanoliter 2000 (World Precision Instruments, Sarasota, Florida, USA). At 6 d post injection, planthoppers were collected for RNA and protein isolation. For PO activity detection, 23 nL of ds*DDC*-RNAs (6 μg μL^-1^) was delivered into nonviruliferous planthopper adults. Planthoppers were collected 3 d after injection.

### Injection of planthoppers with RSV crude preparations

RSV crude preparations were extracted from fifty viruliferous planthopper adults as previously described [32]. A total of 23 nL of RSV crude preparations was injected into the hemolymph of nonviruliferous adults using a Nanoliter 2000. The planthoppers were collected at 4, 6, 8, 10, 12, 14, 16, and 18 DPI for qRT–PCR assay, with six to eight replicates for each group.

### 5’ RLM-RACE

To verify the cleavage of *DDC* by the three vsiRNAs, 5’ RLM-RACE was performed using FirstChoice RLM-RACE (Thermo Scientific; AM1700M) according to the manufacturer’s instructions. Briefly, total RNA was extracted from viruliferous planthoppers and directly ligated to the 5’ RACE Adapter. Random decamers were used to prime cDNA synthesis with reverse transcriptase. Same primers were designed for the binding sites of vsiR-8401 and vsiR-5532. Primers are listed in S6 Table. PCR products were purified, cloned, and sequenced.

### Injection of vsiRNA activator and inhibitor

The activators of vsiR-8401, vsiR-7607, and vsiR-5532 were chemically modified double-stranded oligonucleotides corresponding to the sequences of vsiR-8401, vsiR-7607, and vsiR-5532, respectively (GenePharma). The negative control (NC) sequence for the vsiRNA activator was 5’-UUCUCCGAACGUGUCACGUTT-3’. The inhibitors of vsiR-8401, vsiR-7607, and vsiR-5532 were chemically modified single-stranded nucleotide sequences with reverse complementarity to vsiR-8401, vsiR-7607, and vsiR-5532 (GenePharma). A random sequence of 5’-CAGUACUUUUGUGUAGUACAA-3’ was synthesized as the NC sequence for the vsiRNA inhibitor. The strand (antisense strand for activator) is modified by two phosphorothioates at the 5’ end, four phosphorothioates and a cholesterol group at the 3’ end, and full-length nucleotide 2’ -methoxy modification.

A total of 23 nL of inhibitors or activators of vsiR-8401, vsiR-7607, vsiR-5532 or NC at 250 μM plus an equal volume of RSV crude extracts [53] was delivered into nonviruliferous third-instar nymphs by microinjection using a Nanoliter 2000 system (World Precision Instruments). Planthoppers were collected 6 d after injection. Twenty-three nL of vsiR-7607 activator or NC at 12.5 μM or 23 nL of vsiR-5532 activator, vsiR-8401 activator, or NC at 20 μM were delivered into nonviruliferous third-instar nymphs. Planthoppers were collected 3 d after injection. For PO activity detection, 23 nL of the mixture of three vsiRNA activators or NC at 250 μM was delivered into nonviruliferous planthopper adults, which were collected 3 d after injection.

### Prediction of potential targets of vsiRNAs

Potential targets of vsiRNAs within the RSV genomic RNAs and planthopper mRNAs were predicted using miRanda [29] and RNAhybrid [30] algorithms with cutoff values of -15 and -18 kcal mol^−1^ or by using RNAhybrid (< -18 kcal mol^−1^) only for the minimum free energy (MFE) of the RNA duplex. RSV genomic and complementary genomic RNAs [54] and planthopper genes with 2000-bp upstream and 2000-bp downstream flanking sequences [55] were used for viral and cellular target prediction.

### Validation of potential targets of vsiRNAs

A dual-luciferase reporter assay was performed in *Drosophila* S2 cells for vsiRNA target validation. For viral target validation, the putative target of vsiR-8401 (204 bp sequence from the coding region of *RdRP*), the putative target of vsiR-7607 (214 bp sequence from the coding region of *RdRP*) and the putative target of vsiR-5532 (350 bp sequence from viral RNA1 sequence complementary to the *RdRP* coding region) were cloned and inserted into the luciferase reporter vector psiCHECK2 (Promega; C8021). For cellular target validation, the putative target of vsiR-8401 (131 bp sequence from the open reading frame of *DDC*), the putative target of vsiR-7607 (149 bp sequence from the 3’ UTR of *DDC*) and the putative target of vsiR-5532 (131 bp sequence from the open reading frame of *DDC*) were also cloned and inserted into psiCHECK2 (Promega). Site mutations in the sequences complementary to the “seed” sites of vsiRNAs were generated using a KOD-Plus mutagenesis kit (Toyobo, Osaka, Japan; F0936K). Regular double-stranded RNAs of vsiRNA mimic were synthesized (GenePharma). The sequence of the negative control (NC) for the vsiRNA mimic was 5’-UUCUCCGAACGUGUCACGUTT-3’. S2 cells were cotransfected with recombinant psiCHECK2 plasmids and various concentrations (100 pM, 1 nM, 10 nM, or 50 nM) of vsiRNA mimic or NC using Lipofectamine 3000 (Invitrogen; 11668019). After transfection at 28°C for 24 h, cells were collected to determine the luciferase activity by the Dual-Glo Luciferase Assay System (Promega; E2920) as described previously [21]. Four to six replicates were prepared for each group. The relative activity of Rluc normalized to Fluc activity is presented as the mean ± SE. The primers used in this experiment are listed in S6 Table.

### Verification of vsiRNA precursors

Two types of putative precursor sequences were synthesized in Huigene co., Ltd. (Beijing, China). The ssRNA precursor sequences are 5’- acacauagucagaggaagaauaauuuuauuUUGUUUUCCUCUGGACUUUGUGU-3’ for vsiR-8401, 5’-acacaaagucuggguauaacuggcUUAUAUACCCAGGACUUUGUGU-3’ for vsiR-7607, and 5’-acacaaagucuggguaauaaaauuuucgauaauauaacUAUUUUACCCAGGACUUUGUGU-3’ for vsiR-5532. Each ssRNA sequence was annealed to form the panhandle structure. The sense strands of dsRNA precursor sequences are 5’-gauuauauaaacaaaaacauuUUGUUUUCCUCUGGACUUUGUGU-3’ for vsiR-8401, 5’-guuagauuuauaugauauauguggcUUAUAUACCCAGGACUUUGUGU-3’ for vsiR-7607, and 5’-uguauuguauaguaaaaauauaacUAUUUUACCCAGGACUUUGUGU-3’ for vsiR-5532. The three vsiRNA sequences are in capital letters. S2 cells were transfected with 20 μM ssRNA or dsRNA precursors and incubated at 28°C for 24 h. The RNA levels of each vsiRNA were quantified by qPCR with two pairs of primers and the product sequences were confirmed by Sanger sequencing. Two forward primers F1 and F2 are listed in S6 Table and the reverse primer was from miRcute miRNA qPCR Detection Kit.

### Transcriptome sequencing and analysis

Nonviruliferous third-instar planthopper nymphs after injection with 12.5 μM vsiR-7607 activator for 3 d were collected for RNA sequencing using an Illumina NovaSeq 6000 in Novogene (Beijing, China). Three biological replicates and five insects per replicate were prepared for each group. The quality of raw RNA sequencing reads was evaluated with FastQC. At least 50 million clean reads were produced for each insect sample. Approximately 15 gigabases (Gb) of 150-bp paired-end raw data were generated for each library. Reads of each sample were deposited in the Short Read Archive of the National Center for Biotechnology Information (NCBI) with accession numbers PRJNA943077.

Clean reads of insect samples were mapped to the planthopper genome sequence [55] using HISAT2 [56]. Read counts of the annotated genes were summarized by HTSeq count [57]. EdgeR [58] was used to identify differentially expressed genes (DEGs). The DEGs were determined by setting a 2-fold change and a cutoff q-value less than 0.05.

### PO activity assay

The PO activity of planthopper adults was detected as previously described [32]. In short, 20 adults were homogenized in 100 μL of 10 mM Tris-HCl buffer (pH 8.0), and 75 μL of the supernatant was mixed with 100 μL of L-DOPA (4 mg ml^-1^) (Sigma-Aldrich, Saint Louis, MO, USA; 59-92-7) in a 96-well plate at 27°C for 10 min. Five replicates were prepared. A490 was measured by a SpectraMax Paradigm reader (Molecular Devices, San Jose, CA, USA) every 5 min. The protein concentration of the supernatant was determined by the BCA method using a BCA Protein Quantification Kit (Vazyme, Nanjing, Jiangsu, China; E112). One unit (U) of PO activity was defined as 0.001 ΔOD490 for every milligram protein per 1 min.

### vsiRNA expression pattern in rice with prolonged infection time

Three-week-old seedlings of *Oryza sativa* Wuyujing were inoculated with 20 viruliferous third-instar planthoppers that were trapped in a microcage on a leaf for 4 d. After the viruliferous planthoppers were removed, the leaves were collected for the measurement of RNA levels of RSV *NP* and three vsiRNAs at 4 dpi, 8 dpi and 12 dpi using qPCR.

### Construction of STTM8401 transgenic rice lines and test of RSV resistance

The STTM8401 plasmid was generated following an established protocol [59]. In brief, STTM8401 was constructed with vsiR-8401 binding sites on flanking sides separated by a 48 nt spacer (5’-gttgttgttgttatggtctaatttaaatatggtctaaagaagaagaat-3’) to form an imperfect weak stem-loop. Two primers, STTM8401-forward and STTM8401-reverse (S6 Table), were used to amplify the DNA fragment, which were inserted into *BsaI* and *Eco31I* sites of the vector 35S-pBWA(V)HS (BioRun, Wuhan, Hubei, China; REC10-I). After verified by sequencing, the STTM8401 plasmid was transformed in *O*. *sativa* Nipponbare in BioRun Co., Ltd. Three-week-old seedlings of T2 generation of STTM-8401 lines and wild-type Nipponbare (WT) were inoculated with 10 viruliferous third-instar planthoppers that were trapped in a microcage on a leaf for 7 d. Both insects and leaves were collected for the measurement of RNA levels of RSV *NP* and vsiRNAs using qPCR and protein levels of NP using Western blot at 7 dpi. Data from the STTM-8401 lines with silenced vsiR-8401 were presented and compared with those from WT rice.

### Rice disease incidence assay

Each leave of the WT and STTM8401 rice plants was fed on by 10 viruliferous third-instar planthoppers that were trapped in a microcage for 7 days. Following planthopper removal, disease symptoms were observed in the rice leaves incubated in a greenhouse at 28°C. Five plants per replicate and six replicates were used to calculate the disease incidences.

### Quantifications and statistical analysis

Student’s t-test and one-way ANOVA with Tukey’s multiple comparison test were performed by GraphPad Prism version 8.0. Error bars represented SEM. Four to twelve replicates were prepared and assayed for each group of experiments.

## Supporting information

S1 Fig Interference rates of *Dicer* genes in the samples of Fig 2. The transcript level of *Dicer1* relative to that of *EF2* in nonviruliferous planthoppers after injection of RSV crude preparations with ds*Dicer1*-RNA (A), or ds*Dicer2*-RNA (B), or both ds*Dicer1*- and ds*Dicer2*-RNA (C) for 6 d (n = 7 or 8). Injection of RSV crude preparations with ds*GFP*-RNA was used as control. Values were compared by Student’s t test. ***, P < 0.001.
(TIF)

S2 Fig Interactive effects of each vsiRNA inhibitor on the expression of the other two vsiRNAs. (A) The RNA levels of vsiR-8401 and vsiR-5532 relative to that of U6 snRNA in nonviruliferous planthoppers after injection of the mixture of RSV crude preparations and vsiR-7607 inhibitor for 6 d (n = 8). (B) The RNA levels of vsiR-7607 and vsiR-5532 relative to that of U6 snRNA after injection of the vsiR-8401 inhibitor (n = 8). (C) The RNA levels of vsiR-8401 and vsiR-7607 relative to that of U6 snRNA after injection of the vsiR-5532 inhibitor (n = 7 or 8). NC, negative control. Values were compared by Student’s t test. NS, no significant difference. *, *P* < 0.05. ***, *P* < 0.001.
(TIF)

S3 Fig Real-time PCR verification of the differentially expressed genes from the transcriptomic analyses of the nonviruliferous planthoppers after injection with vsiR-7607 activator. (A) The transcript levels of 17 downregulated genes relative to that of *EF2* (n = 7 or 8). (B) The transcript levels of 7 upregulated genes relative to that of *EF2* (n = 7 or 8). NC, negative control. Values were compared by Student’s t test. NS, no significant difference. *, *P* < 0.05. **, *P* < 0.01. ***, *P* < 0.001.
(TIF)

S4 Fig The transcript levels of the 10 vsiR-7607-downregulated planthopper genes relative to that of *EF2* after injection with activator of vsiR-8401 (A) or vsiR-5532 (B). n = 7 or 8. NC, negative control. Values were compared by Student’s t test. NS, no significant difference.
(TIF)

S5 Fig 5’ RLM-RACE of *DDC* mRNA degradation products. The grey line represents the 5’ and 3’ UTRs of *DDC* mRNA. The blue thick arrow indicates the open reading frame (ORF) of *DDC*. Three black short lines mark the positions of the three vsiRNAs targeting the ORF and 3’UTR of *DDC*. The red arrow denotes the experimentally validated 5’ end, confirmed through 5’RLM-RACE and sequencing, with the adjacent number reflecting the frequency of 5’ RLM-RACE products cleaved at that specific site. The ruler below shows the nucleotide length of the *DDC* mRNA.
(JPG)

S6 Fig Detection of economic traits in WT and STTM8401 lines. (A) Comparative analysis of plant height in mature WT and STTM8401 rice lines, with a scale bar representing 50 cm. (B) The 1000-grain weight of WT and STTM8401 lines. (C-D) Husked grain width (C) and length (D) of WT and STTM8401 lines. Scale bars: 5 mm. n = 10 or 6. The values were reported as the mean ± SE. Different letters indicate a statistically significant difference.
(TIF)

S1 Table Putative viral targets of the three vsiRNAs. (DOCX)

S2 Table Quality of the RNA-seq data for the nonviruliferous planthoppers injected with vsiR-7607 activator or negative control (NC). (DOCX)

S3 Table Downregulated genes in nonviruliferous planthoppers injected with the vsiR-7607 activator. (XLSX)

S4 Table Upregulated genes in nonviruliferous planthoppers injected with the vsiR-7607 activator. (XLSX)

S5 Table Putative planthopper target genes of the three vsiRNAs. (XLSX)

S6 Table Primers used in this study. (DOCX)
