# Supplementary figures and images for: Small interfering RNAs generated from the terminal panhandle structure of negative-strand RNA virus promote viral infection
Source: PLoS Pathog. 2025 Jan 3;21(1):e1012789. doi: 10.1371/journal.ppat.1012789 (PMC11698402; doi:10.1371/journal.ppat.1012789)

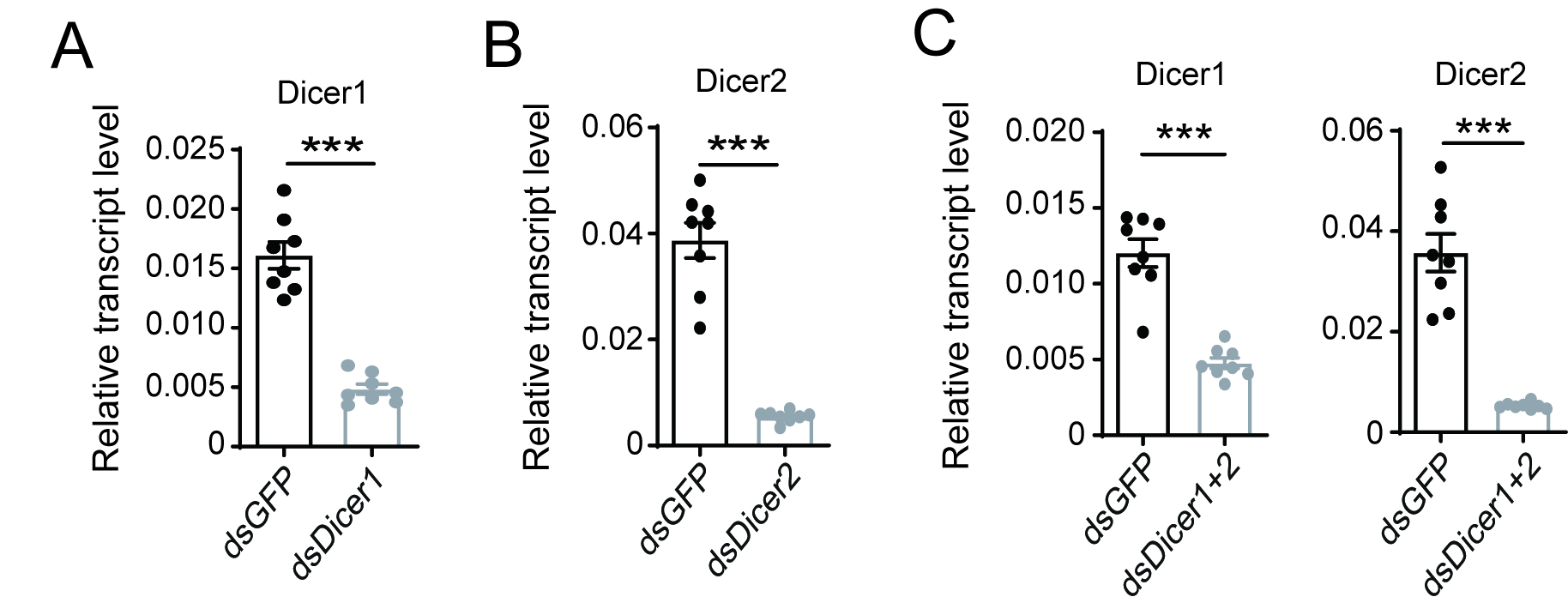

Supplement: S1 Fig — The transcript level of Dicer1 relative to that of EF2 in nonviruliferous planthoppers after injection of RSV crude preparations with dsDicer1-RNA (A), or dsDicer2-RNA (B), or both dsDicer1- and dsDicer2-RNA (C) for 6 d (n = 7 or 8). Injection of RSV crude preparations with dsGFP-RNA was used as control. Values were compared by Student’s t test. ***, P < 0.001. (TIF) [file ppat.1012789.s001.tif]

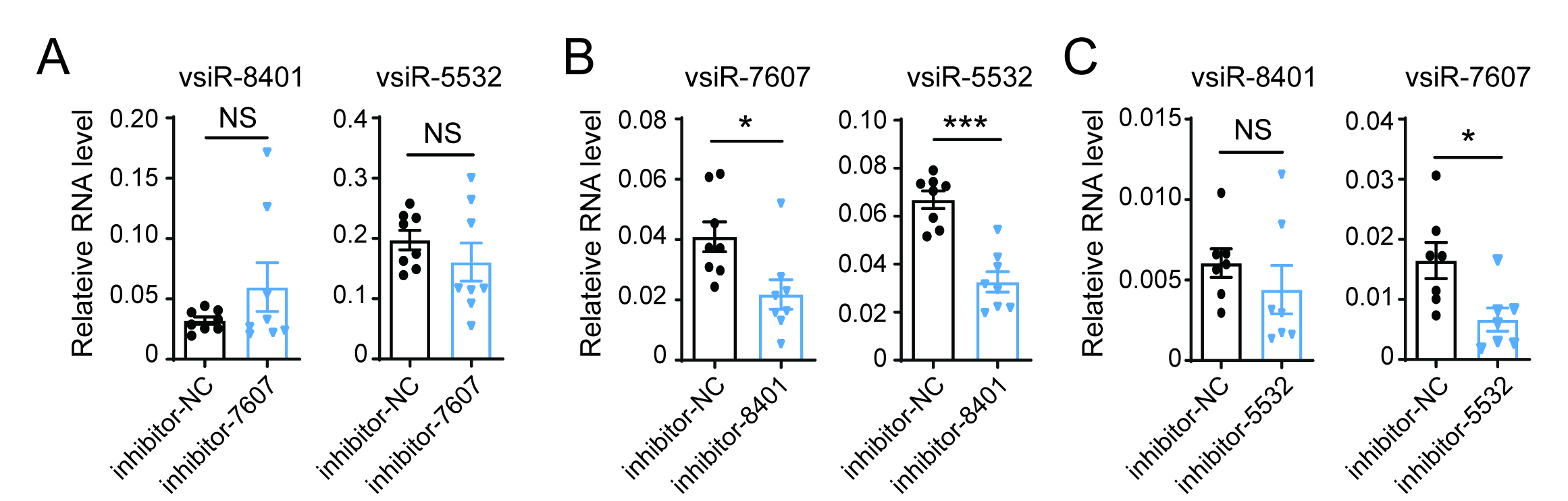

Supplement: S2 Fig — (A) The RNA levels of vsiR-8401 and vsiR-5532 relative to that of U6 snRNA in nonviruliferous planthoppers after injection of the mixture of RSV crude preparations and vsiR-7607 inhibitor for 6 d (n = 8). (B) The RNA levels of vsiR-7607 and vsiR-5532 relative to that of U6 snRNA after injection of the vsiR-8401 inhibitor (n = 8). (C) The RNA levels of vsiR-8401 and vsiR-7607 relative to that of U6 snRNA after injection of the vsiR-5532 inhibitor (n = 7 or 8). NC, negative control. Values were compared by Student’s t test. NS, no significant difference. *, P < 0.05. ***, P < 0.001. (TIF) [file ppat.1012789.s002.tif]

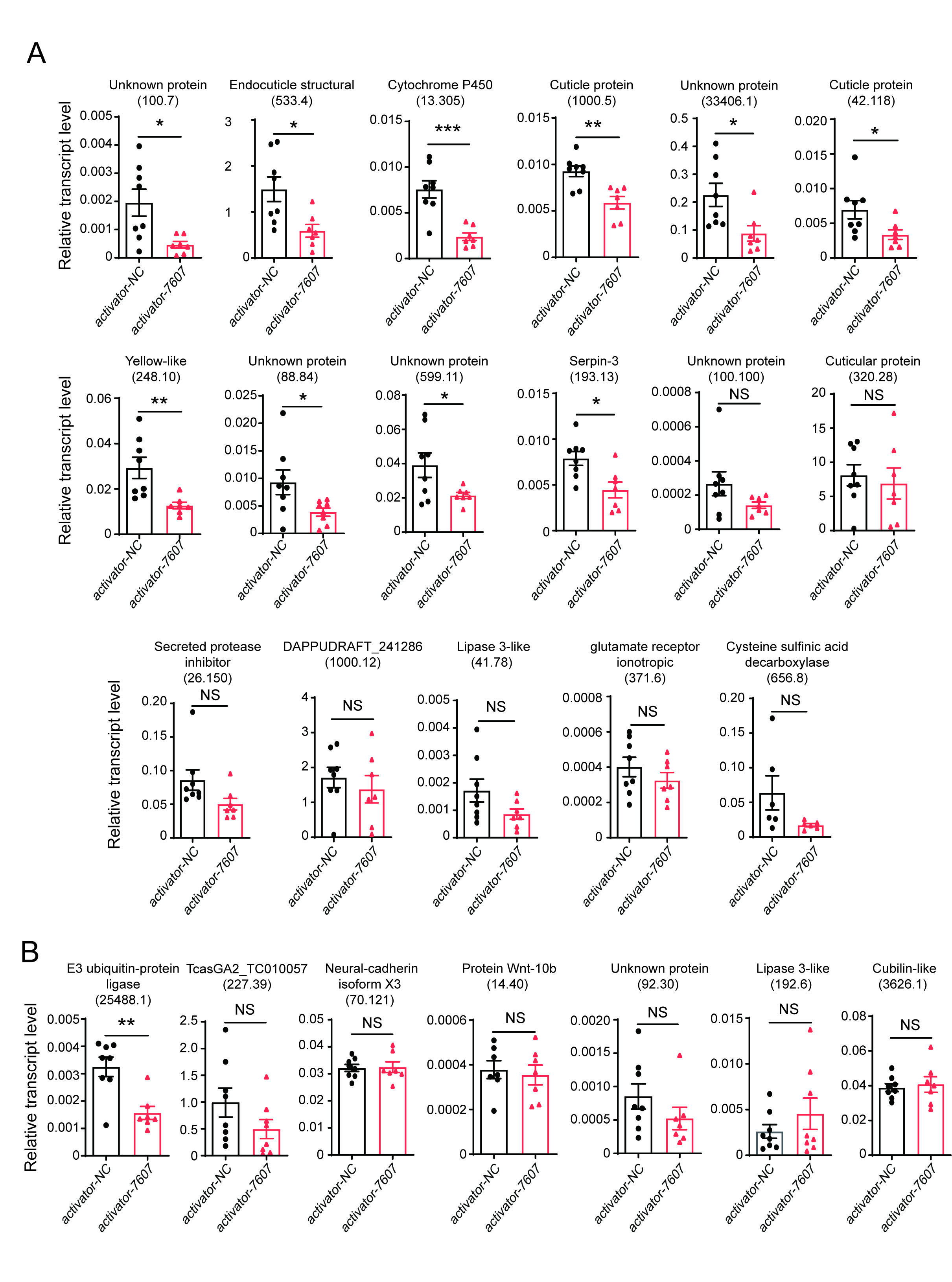

Supplement: S3 Fig — (A) The transcript levels of 17 downregulated genes relative to that of EF2 (n = 7 or 8). (B) The transcript levels of 7 upregulated genes relative to that of EF2 (n = 7 or 8). NC, negative control. Values were compared by Student’s t test. NS, no significant difference. *, P < 0.05. **, P < 0.01. ***, P < 0.001. (TIF) [file ppat.1012789.s003.tif]

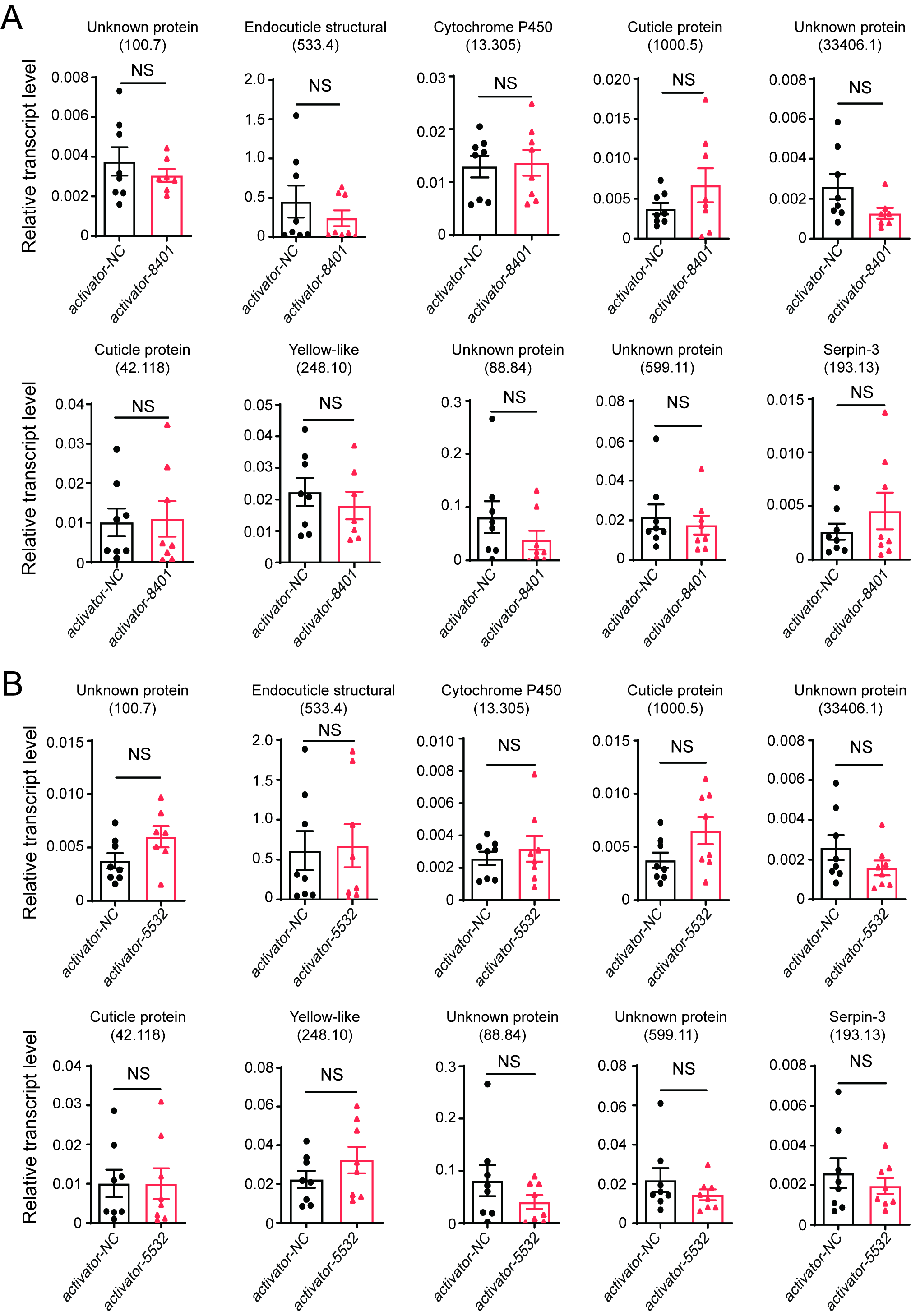

Supplement: S4 Fig — n = 7 or 8. NC, negative control. Values were compared by Student’s t test. NS, no significant difference. (TIF) [file ppat.1012789.s004.tif]

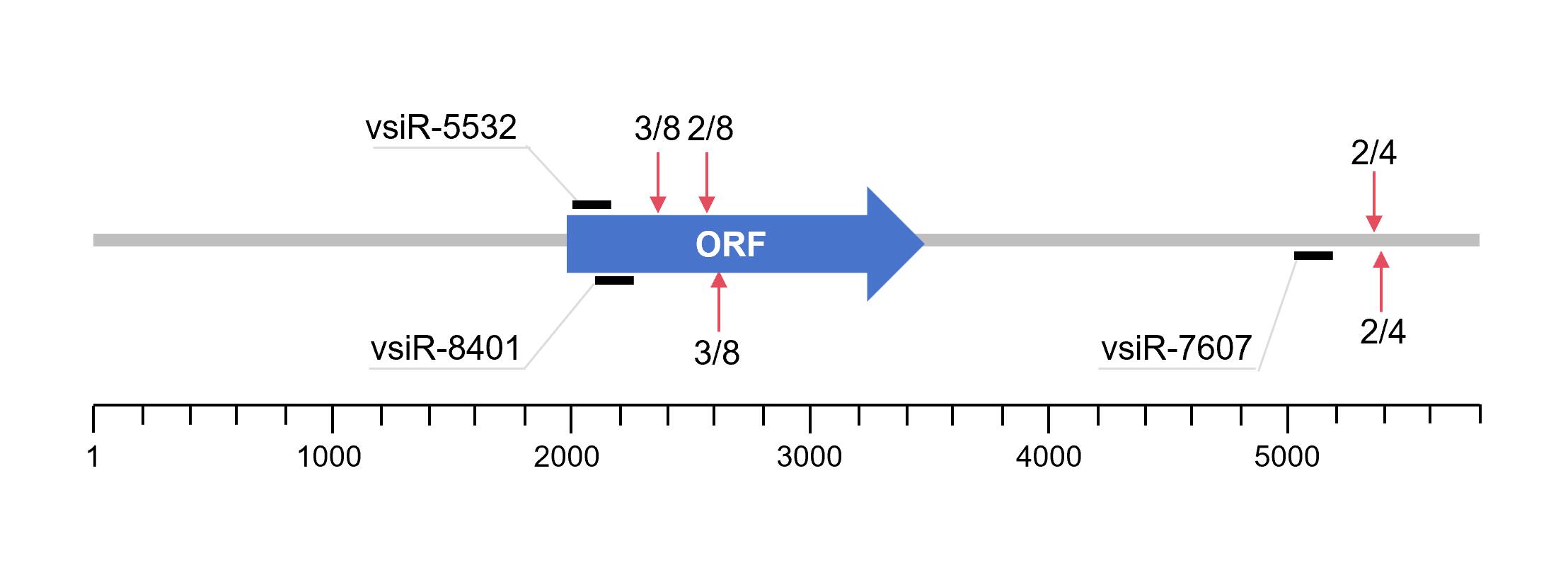

Supplement: S5 Fig — The grey line represents the 5’ and 3’ UTRs of DDC mRNA. The blue thick arrow indicates the open reading frame (ORF) of DDC. Three black short lines mark the positions of the three vsiRNAs targeting the ORF and 3’UTR of DDC. The red arrow denotes the experimentally validated 5’ end, confirmed through 5’RLM-RACE and sequencing, with the adjacent number reflecting the frequency of 5’ RLM-RACE products cleaved at that specific site. The ruler below shows the nucleotide length of the DDC mRNA. (JPG) [file ppat.1012789.s005.jpg]

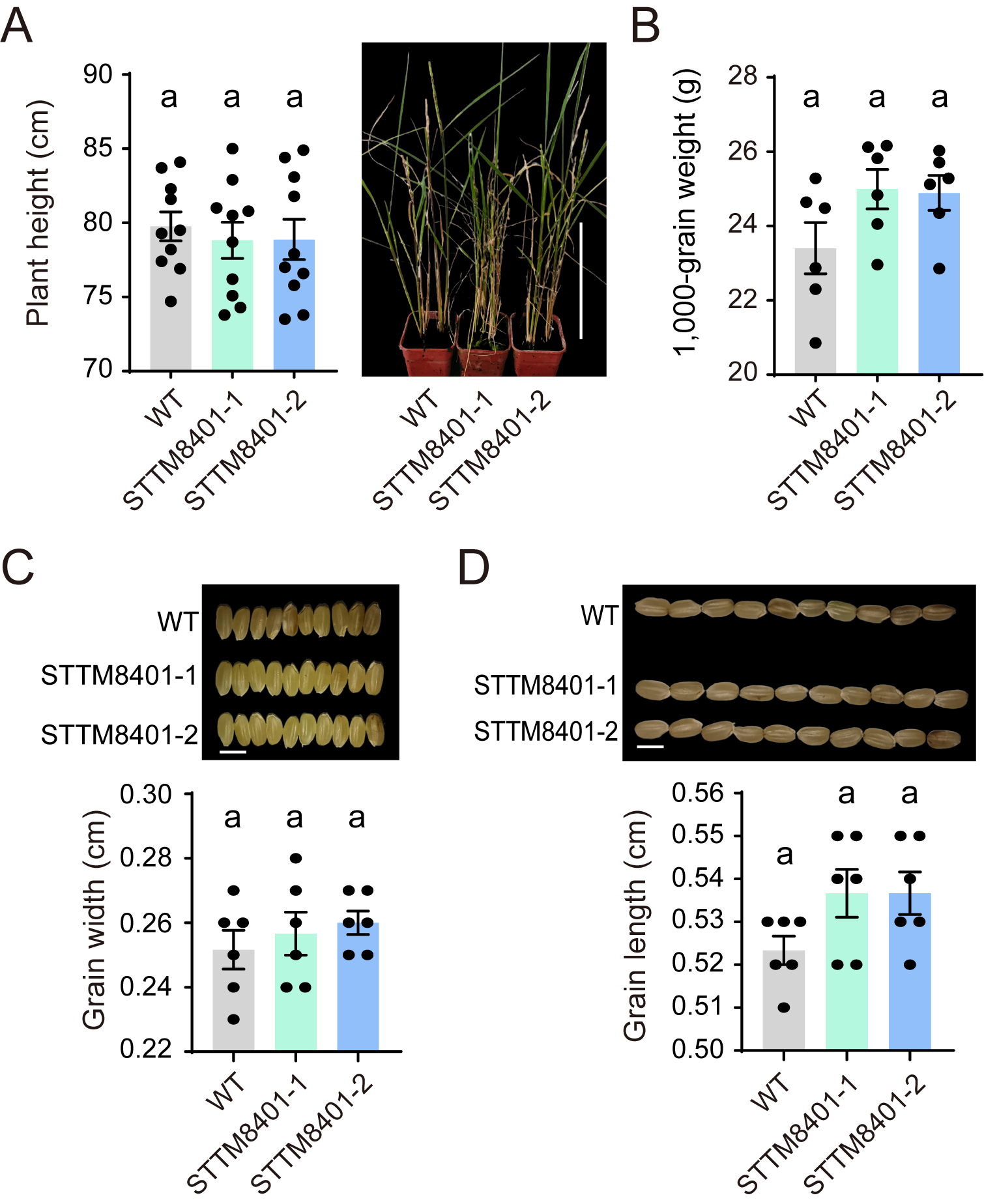

Supplement: S6 Fig — (A) Comparative analysis of plant height in mature WT and STTM8401 rice lines, with a scale bar representing 50 cm. (B) The 1000-grain weight of WT and STTM8401 lines. (C-D) Husked grain width (C) and length (D) of WT and STTM8401 lines. Scale bars: 5 mm. n = 10 or 6. The values were reported as the mean ± SE. Different letters indicate a statistically significant difference. (TIF) [file ppat.1012789.s006.tif]
