# Supplementary material for: Small interfering RNAs generated from the terminal panhandle structure of negative-strand RNA virus promote viral infection
Source: PLoS Pathog. 2025 Jan 3;21(1):e1012789. doi: 10.1371/journal.ppat.1012789 (PMC11698402; doi:10.1371/journal.ppat.1012789)
Supplement: S1 Table — (DOCX) [file ppat.1012789.s007.docx]

**S1 Table. Putative viral targets of the three vsiRNAs**

| vsiRNAs | RNAhybrid  energy (kcal/mol) | miRanda  energy (kcal/mol) | Target site (nt) |
| --- | --- | --- | --- |
| vsiR-8401 | -23.9 | -15.39 | vcRNA1: 7698-7720 |
| vsiR-7607 | -23.8 | -17.76 | vcRNA1: 3215-3235 |
| vsiR-5532 | -20.4 | / | RNA1: 1866-1897 |
|  | -20.2 | / | RNA3: 76-103 |
|  | -19.0 | / | vcRNA4: 164-192 |
|  | -18.6 | / | vcRNA4: 889-921 |
|  | -18.0 | / | vcRNA2: 74-101 |
