# Supplementary material for: Small interfering RNAs generated from the terminal panhandle structure of negative-strand RNA virus promote viral infection
Source: PLoS Pathog. 2025 Jan 3;21(1):e1012789. doi: 10.1371/journal.ppat.1012789 (PMC11698402; doi:10.1371/journal.ppat.1012789)
Supplement: S2 Table — (DOCX) [file ppat.1012789.s008.docx]

**S2 Table. Quality of the RNA-seq data for the nonviruliferous planthoppers injected with vsiR-7607 activator or negative control (NC)**

|  | Raw Reads | Clean Reads | Raw Base (G) | Clean Base (G) | Effective Rate (%) | Error Rate (%) | Q20  (%) | Q30  (%) |
| --- | --- | --- | --- | --- | --- | --- | --- | --- |
| NC-1 | 52,088,863 | 49,858,120 | 15.63 | 14.96 | 95.72 | 0.03 | 96.69 | 91.62 |
| NC-2 | 53,259,994 | 51,170,204 | 15.98 | 15.35 | 96.08 | 0.03 | 96.60 | 91.47 |
| NC-3 | 55,130,697 | 52,833,382 | 16.54 | 15.85 | 95.83 | 0.03 | 97.00 | 92.29 |
| 7607-1 | 53,216,023 | 51,029,023 | 15.96 | 15.31 | 95.89 | 0.03 | 97.04 | 92.41 |
| 7607-2 | 53,867,415 | 51,392,240 | 16.16 | 15.42 | 95.41 | 0.03 | 97.12 | 92.56 |
| 7607-3 | 51,135,466 | 49,153,766 | 15.34 | 14.75 | 96.12 | 0.03 | 96.69 | 91.78 |

Three replicates for the treatment and control groups.
