# Supplementary material for: Small interfering RNAs generated from the terminal panhandle structure of negative-strand RNA virus promote viral infection
Source: PLoS Pathog. 2025 Jan 3;21(1):e1012789. doi: 10.1371/journal.ppat.1012789 (PMC11698402; doi:10.1371/journal.ppat.1012789)
Supplement: S6 Table — (DOCX) [file ppat.1012789.s012.docx]

**S6 Table. Primers used in this study.**

| **Sequence name** | **Sequence (5’ to 3’)** |
| --- | --- |
| Primers for qPCR | |
| U6-q-F | GAACGATACAGAGAAGATTAG |
| U6-q-R | AAATGTGGAACGCTTCACG |
| vsiR8401-q-F | GCGCGCTTGTTTTCCTCTGGACTTTGTGT |
| vsiR7607-q-F | GGCGGTTATATACCCAGGACTTTGTGT |
| vsiR5532-q-F | TATTTTACCCAGGACTTTGTGT |
| vsiR2963-q-F | GCGCCAAATGCCCTGGACTTTGTGT |
| ef2-q-F | GTCTCCACGGATGGGCTTT |
| ef2-q-R | ATCTTGAATTTCTCGGCATACATTT |
| NP-q-F | GGAACAAATGCCAATGCTATC |
| NP-q-R | TGAGACATTTGGGAATAGCTGA |
| Dicer1-q-F | GACAAACGACAACACTGCCC |
| Dicer1-q-R | TCACTTATGGCATGGCCGTT |
| Dicer2-q-F | ACGCTTCACGTGCAAATGTC |
| Dicer2-q-R | CAAGCCTTCTCGGGTGTCAT |
| DDC-q-F | CGAAGAACCCGATAAGTGGC |
| DDC-q-R | ATTCAGTACAGGCGGGACTG |
| Contig100.100-q-F | GGTTGTCGTGAGTTTTGCGG |
| Contig100.100-q-R | CACCATCCAACTCGCTGAGA |
| Contig100.7-q-F | GGATCTGAAGGACGGCGAAA |
| Contig100.7-q-R | CTTCCACGTTTGCTATGGCG |
| Contig320.28-q-F | TTGTCTTCGGAGTAGCCCTG |
| Contig320.28-q-R | CGGTGTGCTCGTCTTTCACT |
| Contig26.150-q-F | ACCACCTGCAACATTCGCTA |
| Contig26.150-q-R | GTTCGCACTTGTCGTCATCG |
| Contig533.4-q-F | GTGTAGCTGCCTTGACCGTA |
| Contig533.4-q-R | TGCCTGTTTCGTAGCTGTATT |
| Contig13.305-q-F | TAGCCTCTGAACTGCCAGGG |
| Contig13.305-q-R | GTTTCTGCTGTCTGTAAGCGT |
| Contig1000.12-q-F | GGTGATGATCGCCGTGCTAA |
| Contig1000.12-q-R | GGTGTGCTCGTCCTTGACTG |
| Contig1000.5-q-F | ATATAAAGCGAAGCCCCGCC |
| Contig1000.5-q-R | AGGCACGACACTAGAGCGAT |
| Contig33406.1-q-F | CTTGGACACACCACAGGTCA |
| Contig33406.1-q-R | AGCTGTGTGCGTACTGAGTC |
| Contig42.118-q-F | AGGGAGAGTACTCGGTGGTC |
| Contig42.118-q-R | ATCCGTGATGGTAGACGGGA |
| Contig41.48-q-F | ACGTACGCGGCAATCTGTAT |
| Contig41.78-q-R | GCCATGGTGGTACCCATTGA |
| Contig248.10-q-F | TAGCCTACGACCCGGAAAGA |
| Contig248.10-q-R | CGGGTTGACCGATTGTCGTA |
| Contig599.11-q-F | AAAACGTTGTTTCGGTGGGC |
| Contig599.11-q-R | CACTGAAGCTGGTTGCTGGA |
| Contig88.84-q-F | GGCCAGTACACACTGTTGGA |
| Contig88.84-q-R | CGTGACCTGACTTGGTGACA |
| Contig45.125-q-F | ATGATTCTGAAAGTAGCTGATAATC |
| Contig45.125-q-R | GTCTCTCCAACAGACCACC |
| Contig371.6-q-F | CAATATGGCGACGTGCGTTC |
| Contig371.6-q-R | CGTTCCTCGCCCAGTTTAGT |
| Contig193.13-q-F | GGAGTCTCGTGTGATGGACC |
| Contig193.13-q-R | CCAGGGCGTAAAAAGGACGA |
| Contig656.8-q-F | AAGCGACACGACCACTTCAT |
| Contig656.8-q-R | TCACAAAGTACGGATGGCCC |
| Contig25488.1-q-F | AGAATCAGCCCGCACTGAAA |
| Contig25488.1-q-R | GGTTATGCGCCAGCAGATTG |
| Contig227.39-q-F | CGTTGTCCAGGGCGAATACA |
| Contig227.39-q-R | TTAGCGATGGGTTCGTTGGG |
| Contig70.121-q-F | ACTACATGGGTCAAGCACCG |
| Contig70.121-q-R | CCTCGTTGAGCCTGAACTGT |
| Contig14.40-q-F | ATTTTGGTCGTCTCGCGGTA |
| Contig14.40-q-R | GATCCGTTGCCGAGATTGGA |
| Contig92.30-q-F | ACGGGCGTGTTGAAAAATCG |
| Contig92.30-q-R | TTCTGGAAGCGAGATGCGTT |
| Contig192.6-q-F | TAGCGTTCGACCATTGCTCA |
| Contig192.6-q-R | AAAGGCCCATTCCTTGTGCT |
| Contig3626.1-q-F | TTCAGTCACCCGAATCAGCC |
| Contig3626.1-q-R | ACCGTAGGCTGAGAGGTAGG |
| RdRP-8401bs-RIP-F | GGAGAGGTTCATTCGATGATTCC |
| RdRP-8401bs-RIP-R | TTCAGATGGCCCATCAAGAC |
| RNA1-5532bs-RIP-F | AGCTCCTTGTGTGCTAGTTCTC |
| RNA1-5532bs-RIP-R | TACAAGAAGTCGTGGCAGATG |
| DDC-8401bs-RIP-F | TCGGCCACCAATTGAGTG |
| DDC-8401bs-RIP-R | GATGTACAGTAGCCTAC |
| DDC-7607bs-RIP-F | GCATTAACTTAGTCATAGAC |
| DDC-7607bs-RIP-R | TTGAGCTGTTACGATGG |
| DDC-5532bs-RIP-F | GGCACAAGAAATGGAGC |
| DDC-5532bs-RIP-R | GAAGAACTCTCCTGTCTC |
| Primers for RNAi | |
| GFP-dsRNA-F | CACAAGTTCAGCGTGTCCG |
| GFP-dsRNA-R | GTTCACCTTGATGCCGTTC |
| Dicer1-dsRNA-F | CACCATGTCCAATGCCAACG |
| Dicer1-dsRNA-R | CACTCGATCAGATAGGCACCA |
| Dicer2-dsRNA-F | CCAACCACCATGCGATCCTA |
| Dicer2-dsRNA-R | CGCCAACACGGATAGAGGTT |
| DDC-dsRNA-F | GTCGATTCGCATTACAGACTG |
| DDC-dsRNA-R | TGTGTAACGGGAACAGATAGC |
| GFP-T7-F | TAATACGACTCACTATAGGCACAAGTTCAGCGTGTCCG |
| GFP-T7-R | TAATACGACTCACTATAGGGTTCACCTTGATGCCGTTC |
| Dicer1-T7-F | TAATACGACTCACTATAGGCACCATGTCCAATGCCAACG |
| Dicer1-T7-R | TAATACGACTCACTATAGGCACTCGATCAGATAGGCACCA |
| Dicer2-T7-F | TAATACGACTCACTATAGGCCAACCACCATGCGATCCTA |
| Dicer2-T7-R | TAATACGACTCACTATAGGCGCCAACACGGATAGAGGTT |
| DDC-T7-F | TAATACGACTCACTATAGGGTCGATTCGCATTAC |
| DDC-T7-R | TAATACGACTCACTATAGGTGTGTAACGGGAAC |
| Primers for expression plasmid construction | |
| RdRP-8401bs-F | GAATTGAGGAGCTAGATGGTG |
| RdRP-8401bs-R | TCTCTGCACCATGTCTTCAG |
| RdRP-7607bs-F | AGTAATGTGTTCTTCTCCTTATAC |
| RdRP-7607bs-R | TCAATACTAGGAGCCACAGC |
| RNA1-5532bs-F | TGCAAGCACATATGGGGTTTTG |
| RNA1-5532bs-R | ACCCTGTCTCAGGCACCTAT |
| DDC-8401bs-F | GAACACTATTCTCTATTTACAGGG |
| DDC-8401bs-R | CAACGGTTGGAAGAACTCTC |
| DDC-7607bs-F | ACCAAGGTTCTCTCAGTTCC |
| DDC-7607bs-R | TATTTGAGCTGTTACGATGGAG |
| DDC-5532bs-F | GAACACTATTCTCTATTTACAGGG |
| DDC-5532bs-R | CAACGGTTGGAAGAACTCTC |
| RdRP-8401bs-Mut-F | GCATAGTTGTCCAAGACCTGAACCUUUAACCG |
| RdRP-8401bs-Mut-R | AACATCGGAATCATCGAATGAAC |
| RNA1-5532bs-Mut-F | CATCTCATGGATCATCTGCCACGACTTCTTG |
| RNA1-5532bs-Mut-R | ATTGGCCTGACCAGGACACAAGTCAGAAA |
| DDC-8401bs-Mut-F | GTACATTGGCCAATACTTCCUUCUGC |
| DDC-8401bs-Mut-R | TCCACCATTGATTTCGCGAATTCG |
| DDC-7607bs-Mut-F | GAGACTACATTCAGTCTCACACAGATAT |
| DDC-7607bs-Mut-R | TGTAAATTTTGTTACTATGAGTCTATGAC |
| DDC-5532bs-Mut-F | TCGCGAAATCAATGCACCTCTAC |
| DDC-5532bs-Mut-R | ATTCGCTAAACTCTTTCTGCTCC |
| Primers for RLM-RACE | |
| 5’outer-8401/5532 | TTCACTGGCAGTTCCCTGAA |
| 5’inner-8401/5532 | TGCTGGATATGAATTGGCTG |
| 5’outer-7607 | TTGGATACTCACTCTCTGAT |
| 5’inner-7607 | CACACTAATCTCTTACGGTACT |

^a^ F, forward primers; R, reverse primers.
